# Pre-Sowing E-Beam and X-Ray Irradiation of Wheat Seeds to Enhance Yield and Improve Phytopathogenic Status of Crops

**DOI:** 10.3390/plants15121806

**Published:** 2026-06-11

**Authors:** Natalya Chulikova, Yana Zubritskaya, Anna Malyuga, Ulyana Bliznyuk, Polina Borshchegovskaya, Aleksandr Nikitchenko, Victoria Ipatova, Dmitry Yurov, Grigorii Krusanov, Maria Chibisova, Sergei Goloschapov, Alexander Chernyaev, Tatyana Saltykova, Igor Rodin, Elena Kozlova

**Affiliations:** 1 Siberian Federal Scientific Center of Agro-Biotechnologies, Russian Academy of Sciences, Novosibirsk Oblast, 630501 Krasnoobsk, Russia; natalya-chulikova@yandex.ru (N.C.); anna_malyuga@mail.ru (A.M.); sergei-goloshapov@yandex.ru (S.G.); 2 Department of Physics, Lomonosov Moscow State University, GSP-1, 1-2 Leninskiye Gory, 119991 Moscow, Russia; zubritckaia.iv18@physics.msu.ru (Y.Z.); alexeevapo@mail.ru (P.B.); nikitchenko.ad15@physics.msu.ru (A.N.); chibisova.ms20@physics.msu.ru (M.C.); a.p.chernyaev@yandex.ru (A.C.); 3 Skobeltsyn Institute of Nuclear Physics, Lomonosov Moscow State University, GSP-1, 1-2 Leninskiye Gory, 119991 Moscow, Russia; ipatova.vs15@physics.msu.ru (V.I.);; 4 State Research Center-Burnasyan Federal Medical Biophysical Center of Federal Medical Biological Agency, Zhivopisnaya Street, 46, 123098 Moscow, Russia; krusanov@physics.msu.ru; 5 Department of Medical and Biological Physics, Sechenov First Moscow State Medical University, 8-2 Trubetskaya Str., 119991 Moscow, Russia; saltykova_t_s@staff.sechenov.ru (T.S.); igorrodin@yandex.ru (I.R.); waterlake@mail.ru (E.K.); 6 Department of Chemistry, Lomonosov Moscow State University, GSP-1, 1-3 Leninskiye Gory, 119991 Moscow, Russia; 7 Department of Analytical Chemistry, Lomonosov Institute of Fine Chemical Technologies, MIREA–Russian Technological University, Vernadsky Avenue, 78, 119454 Moscow, Russia

**Keywords:** pre-planting irradiation, wheat seeds, e-beam, X-rays, fungal diseases, GEANT4 computer simulation, optimal dose range, radiation hormesis

## Abstract

The two-year research involving laboratory and field studies supported by Geant4 computer simulation is aimed at determining the optimal parameters of 1 MeV accelerated electrons and 80 keV X-ray pre-planting irradiation of wheat seeds in order to find the optimal dose range which increases the crop yield while making wheat plants more resistant to fungal diseases caused by species of the genus *Septoria*. During the laboratory studies we measured the germination rate and biometric properties of plants, as well as the type, number, and average diameter of fungi found in the irradiated and non-irradiated seeds after irradiation with electrons and X-rays with the dose range 2–1000 Gy. Following the laboratory studies showing that the doses exceeding 30 Gy decreased the germination rate of wheat, field studies evaluated the impact of pre-planting irradiation with the doses in the range of 5–30 Gy on the wheat productivity and the rate of fungal diseases in wheat plants grown from irradiated and non-irradiated seeds. It has been found that the dose range 5–15 Gy is more preferable for pre-planting wheat irradiation, both for e-beam and X-rays, since it increases the crop yield while making wheat plants more resistant to fungal diseases caused by species of the genus *Septoria*. The X-ray dose of 15 Gy is found to be the most effective since it increased the yield up to 40% and also suppressed the *Septoria* glume blotch up to 40%. Since seed irradiation requires a particularly delicate approach given that the goal of irradiation is not only to reduce the rate of fungal diseases in the plants but also to increase the crop yield without detriment to the soil and the plant itself, consistency of dose uniformity across the seeds during pre-planting irradiation ensures the high reliability and repeatability of the irradiation effect. Our approach to irradiation planning with the use of Geant4 computer simulation allows us to precisely estimate the dose distribution in individual seeds and the distribution of radiation-chemical yield of radicals occurring as result of radiolysis in order to predict the effect of pre-planting irradiation and select the optimal irradiation parameters for maximizing the yield and crop quality.

## 1. Introduction

Wheat, with a total world production exceeding 770 million tons, is one of the most widely grown cereal crop which is used in food manufacturing and as fodder [1]. The production of wheat, however, is threatened by a range of environmental and biological factors, including deterioration of hydrothermal conditions [2], loss of soil fertility [3], extensive spread of traditional crop pests and microorganisms, and the emergence of introduced pathogens. Agricultural challenges in the constantly changing environmental conditions are exacerbated by the extensive use of pesticides, leading to loss of biodiversity, changes in the composition of soil microorganisms, and phytopathogen resistance to pest control treatment [4,5].

Being under a constant pressure from the market experiencing a growing demand in wheat, agricultural industry calls for enhanced treatment methods which would secure a greater yield of high-quality wheat crops. Traditional agricultural practices, including crop rotation, are supplemented by more sophisticated biological, chemical, and physical methods [6] to ensure a consistent growth in the yield despite the adverse conditions.

The use of biofertilizers to increase the availability and uptake of mineral nutrients by plants and crop protection through the introduction of biopesticides, pathogen antagonistic microorganisms, and other biological control agents, increase the yield to a certain degree [7,8,9,10]. However, the use of biofertilizers cannot be regarded as a reliable method for increasing the crop yield since it is highly dependent on environmental factors, such as soil temperature, moisture, and pH [11,12,13,14].

Soil fertility is commonly enhanced by the use of chemical fertilizers containing nitrogen, potassium, and phosphorus. The continuous use of chemical fertilizers, however, leads to a gradual deterioration of quality and fertility of agricultural soils as well as the reduction of soil biodiversity. It should also be noted that the accumulation of heavy metals and other substances in plants can pose a danger to the health of consumers [15,16]. While chemical methods of plant protection using fungicides, insecticides, and herbicides are beneficial since they increase crop yield, they cannot be regarded as a permanent solution to various agricultural problems due to the fact that they increase the resistance of phytopathogens in crops and soil and cause a negative impact on the environment and human health [17,18,19].

The insufficiency of biological and chemical methods can be compensated by the environmentally friendly physical methods for pest control and quality assurance including temperature manipulation and ultrasound and electromagnetic treatment as well as gamma irradiation used to suppress phytopathogens in seed material while enhancing the biometric properties of wheat and crop yield [20,21,22,23].

Pre-sowing irradiation of seed material, provided that irradiation parameters are appropriately selected, allows us to decrease chemical load on plants and soil and, at the same time, effectively stimulates plant growth, suppresses the growth of phytopathogens, and enhances plant resistance to pathogens. Depending on the irradiation goal, effective doses can range from units to hundreds of Grays. The effect of treatment can vary depending on the irradiated crop. For instance, for increasing the height and number of shoots of rice, the effective dose of gamma irradiation is 50 Gy, and for the crop mung—about 200 Gy [24]. Gamma irradiation with the doses of 0.25–5 Gy can be used to support okra growth [25], but for coffee beans, a dose of 50–100 Gy is required to increase the viability of seedlings [26]. A positive effect for barley seeds at gamma irradiation is observed at the doses of 16–20 Gy [27], while high-energy proton beam at the dose of 3 Gy stimulates the growth of seedlings and reduces the harmful effects of salt stress on them [28]. In the case of surface electron beam irradiation of barley seeds with electron energies in the order of hundreds of keV, seed germination increase can be observed at irradiation up to 5000 Gy [29]. Thus, the result of pre-sowing irradiation is determined not only by the type of crops but also by the type of irradiation and its energy spectrum.

The major fungal pathogens associated with wheat seeds include species of the genera *Alternaria*, *Fusarium*, *Septoria*, *Bipolaris*, *Aspergillus*, and *Penicillium*, which are responsible for seed-borne diseases, root rot, leaf and glume blotch, and post-harvest mold. These pathogens can significantly reduce germination rate, biomass, and grain yield, while also compromising grain quality through mycotoxin accumulation.

To ensure the purity of seed material from phytopathogens, higher radiation doses are required. For example, seeds of lotus and corn require the doses 1700–10,000 Gy, depending on the genus of mycopathogens, for the elimination of fungi [30,31]. Thus, *Alternaria* spp. is eliminated from wheat with X-ray irradiation at 150 Gy [32], while *Aspergillus* spp. requires the dose of 2500 Gy to be eliminated from cannabis [33], *Botrytis cinerea* or *Monilinia fructicola* require gamma radiation at the dose of 1000 Gy to be eliminated from peach [34]. In contrast, being highly radiation-resistant, *Fusarium moniliforme* persists on corn seeds up to the dose of 30,000 Gy [35].

The difference in the effect of pre-sowing irradiation on germination, yield, and phytopathogenic status of crops is determined by linear energy transfer (LET) values, which depend on both the type of radiation and its energy spectrum. Thus, the approach to establishing optimal irradiation parameters should take into account the irradiation dose uniformity and LET distribution throughout the irradiated seeds.

Since phytopathogens can be predominantly found in the surface layers of seeds, radiation targeting the surface layers in order to suppress phytopathogens inevitably damages critical structures responsible for seed growth. Therefore, establishing the optimal dose range and the method for efficient irradiation of seeds [36] should be flexible enough to factor in the diverse properties of seed varieties, such as depth at which critical growth structures are located, as well as type and contamination of phytopathogens which can be found in seeds.

While a few decades ago gamma irradiation generated by ^60^Co and ^137^Cs was a prevalent irradiation source for the treatment of crops, now, irradiation with accelerated electrons dominates the scene since electron accelerators allow to vary the energy and penetration depth of electrons to achieve a high-precision irradiation of the layers affected by pathogenic microorganisms and targeting biological structures responsible for plant germination and crop yield. According to the IAEA recommendations, the transition to low-energy accelerated electrons and low-energy bremsstrahlung irradiation sources is relevant since these sources can achieve high dose rate which increases the speed of pre-sowing seed treatment. Moreover, low-energy irradiation sources are convenient for integration into the manufacturing facility compared to radioisotopes [37,38].

Considering that low-energy electrons and photons can cause different effects on yield and crop diseases caused by a wide variety of pathogens, a key challenge is to find the most effective irradiation method of pre-sowing irradiation of wheat seeds which, on the one hand, increases the crop yield and, on the other hand, decreases the ratio of diseased plants. This study compares the impact of low-energy accelerated electrons and low-energy X-rays at different doses on wheat productivity and its phytopathogenic status based on laboratory experiments and field studies conducted for two years.

## 2. Materials and Methods

### 2.1. Research Stages

The two-year study from 2023 to 2024 was conducted on wheat seeds of the Novosibirskaya 29 variety in two repeatable cycles consisting of pre-sowing irradiation and subsequent laboratory studies and field studies. The number of seeds in two-year experiment was 3860; 1200 seeds were intended for laboratory experiments and 1330 seeds for field experiments. Wheat seeds were put in 60 mm-long, 40 mm-thick zip-lock polyethylene bags in a single layer with a width of 3 mm, 30 seeds in each bag for laboratory studies and 95 seed in each bag for field studies for further irradiation. The non-irradiated seeds were stored in bags under the same conditions as the irradiated ones.

Each year before planting, the seeds intended for irradiation were divided into two equal portions; one portion was irradiated with 1 MeV accelerated electrons from one side and the other one was exposed to 80 keV X-rays from one side. In 2023, the seeds used in the laboratory studies were irradiated with doses in the range of 4–150 Gy. In 2024, the wheat seeds were irradiated with doses ranging 10–1000 Gy in order to investigate the effect of higher doses on phytopathogenic status of the seeds. Since laboratory studies revealed that the doses above 50 Gy decreased the seed germination rate, in the field studies in 2023 and 2024, it was feasible to use the doses in the range of 5–30 Gy, bearing in mind that the main goal of the study was to find the optimal pre-sowing irradiation method for wheat seeds that would increase the percentage of the crop unaffected by fungal diseases. Table 1 presents the breakdown of the number of seeds and the irradiation doses used in the laboratory and field studies in 2023 and 2024.

In 2023 and 2024, the laboratory experiments involved the estimation of seed germination rate and phytopathogenic analysis of fungi on wheat seeds, consisting of the measurement of number of fungi colonies and their average diameter. The field studies included the monitoring of seed growth, biometric properties, and yield of wheat crop whose seeds were planted in Siberian soil with a typical phytopathogenic load.

Since wheat seeds contain both germination structures and nutrients that could be affected during pre-sowing irradiation, it was of interest to simulate the irradiation process in order to map the dose distribution in the seeds irradiated with accelerated electrons and X-rays. Geant4 computer simulation was performed to assess linear energy transfer (LET) distribution in seeds during electron and photon irradiation, which is an important indicator of the effect of irradiation on biological objects. Geant4-DNA computer simulation involving the calculations of the distribution of radiation-chemical yield of free radicals initiated by irradiation throughout the volume of the irradiated wheat seeds allowed to understand the mechanisms behind the positive effect of low-energy e-beam and X-ray irradiation on the wheat productivity and reduction of the fungal disease rate.

### 2.2. Wheat Seeds Samples

The laboratory and field studies were conducted on wheat seeds of the Novosibirskaya 29 variety, with protein content in the grain of 16.9% and the gluten content of 36.8% [39,40] naturally infected with phytopathogenic fungi. The Novosibirskaya 29 wheat with a yield amounting to over 50 quintals per hectare, which is considered high in the given climatic and soil conditions, has a growing season of 70–78 days. In total, 3860 wheat seeds were monitored for two years to estimate the impact of pre-sowing irradiation on biometric properties, crop yield, and contamination rate of harvested wheat.

### 2.3. Irradiation Method

#### 2.3.1. E-Beam Irradiation

**Figure A1:**
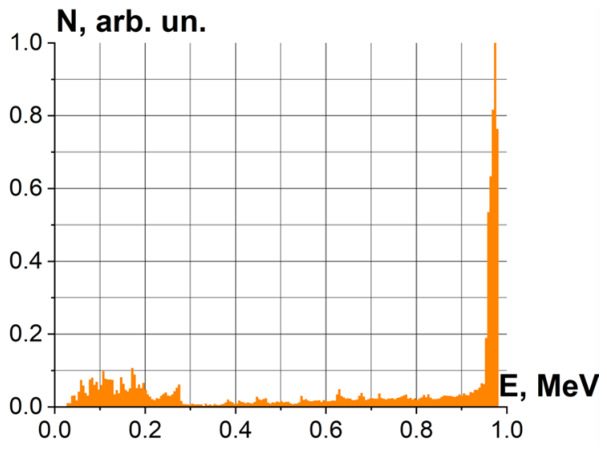
Energy spectrum of electron beam generated by electron accelerator UELR-1-25-T-001.

The wheat seeds were irradiated with accelerated electrons generated by UELR-1-25-T-001 continuous electron accelerator with the maximum energy of 1 MeV and the average beam power of 25 kW designed by Scobeltsyn Research Institute of Nuclear Physics at Moscow State University (Moscow, Russia). The energy spectrum of the beam is shown in Figure A1. The polyethylene bags filled with wheat seeds were put on a 35 cm-long, 5 mm-thick duralumin plate placed at a distance of 12 cm from the beam output and were irradiated with the dose rate of (1.01±0.10) Gy/s. The non-irradiated seeds were stored in bags under the same conditions as the irradiated ones. The parameters of e-beam irradiation are shown in Table A3.

#### 2.3.2. X-Ray Irradiation

**Figure A2:**
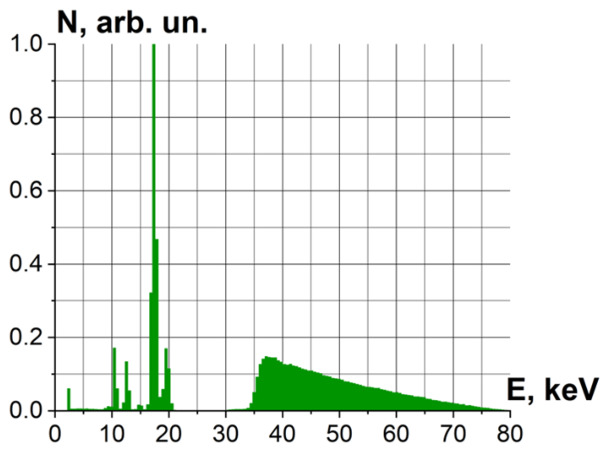
Photon energy spectra generated by RAP-100 X-ray apparatus with a Mo 1VRV23-100 X-ray tube at the voltage of 80 kV.

X-ray irradiation of wheat seeds was generated by X-ray apparatus RAP-100 with a 1BPV 23-100 X-ray tube (Burnazyan FMBC, Moscow, Russia) and a molybdenum anode set to perform at $U_{A}=80\;kV$ and I=5 mA. The X-ray energy spectrum is shown in Figure A2.

Wheat seeds put in the polyethylene bags in a single layer were X-ray irradiated at the dose rate of (0.48±0.03) Gy/s, one bag after another, at the tray located at 12 cm from the beryllium window of the X-ray tube. The non-irradiated seeds were stored in bags under the same conditions as the irradiated ones. The parameters of X-ray irradiation are shown in Table A1.

### 2.4. Dosimetry Control

The doses absorbed by individual wheat seeds during electron beam irradiation and X-ray irradiation were determined using a two-stage dose conversion method. At first, the doses absorbed by the Fricke dosimetry solution were measured during e-beam irradiation and X-ray irradiation performed using the parameters applicable to our irradiation method of wheat seeds.

Using Geant4 computer simulation we calculated the dose conversion coefficient, which is the ratio of the dose absorbed by water phantoms simulating wheat seeds to the dose absorbed by Fricke dosimetry solution, obtained using the same simulation parameters separately for e-beam irradiation and X-ray irradiation. Knowing the experimental doses absorbed by the Fricke dosimetry solution allowed us to determine the doses absorbed by the real wheat seed samples using the dose conversion coefficients for e-beam $K_{e}$ and X-ray irradiation $K_{X}$.

The Fricke solution containing 0.4 M sulphuric acid, 0.2 mM ferric ammonium sulphate, and 0.26 mM potassium chloride was prepared using ultrapure water and thoroughly mixed to saturate the solution with air according to the methodology described in [41].

The height of the Fricke solution was 2.2 mm, the volume of the dosimetry solution put in 35 mm Petri dishes for subsequent irradiation was 1.88 mL. Considering the working range of the standard Fricke solution, the total amount of irradiation sessions was six and they lasted 60–360 s, with a 60 s increment. Electron beam irradiation of the Fricke solution was performed at the beam current $I_{e,0}=0.2\;\mu A$, which corresponded to the beam current applied to the real wheat seed samples in our experiment. X-ray irradiation sessions lasted 120–960 s, with a 2 min increment with eight irradiation sessions in total. X-ray irradiation was performed at the voltage $U_{A}=80\;kV$ and current $I_{X,0}=5\;mA$, which corresponded to the X-ray tube parameters applied to the real wheat seed samples in our experiment.

The dose *D* in Gy absorbed by Fricke solution was calculated using the following formula:

(1) $$D=\frac{k\Delta S(Fe^{3+})}{\rho l\varepsilon G(Fe^{3+})}$$

where ε = 2160 L/(M cm) is the molar extinction coefficient of ${\mathrm{Fe}}^{3+}$ ions, l=1 cm is the optical path length, $k=6.022\times 10^{23}\times 100\times 1.602\times 10^{-19}\;=\;9.65\times 10^{6}$ is a dimensionless unit conversion factor, $\rho =1.024\;g/cm^{3}$ is the density of the dosimetry solution, and ΔS is the change in optical absorbance of the dosimetry solution measured after irradiation at a wavelength of λ=304 nm on a UV-3000 spectrophotometer (TM ECOVIEW, St. Petersburg, Russia) [42]. The radiation-chemical yield G(${\mathrm{Fe}}^{3+}$) is 14.4 ions/100 eV for photons and 15.6 ions/100 eV for electrons [43,44].

**Figure 1:**
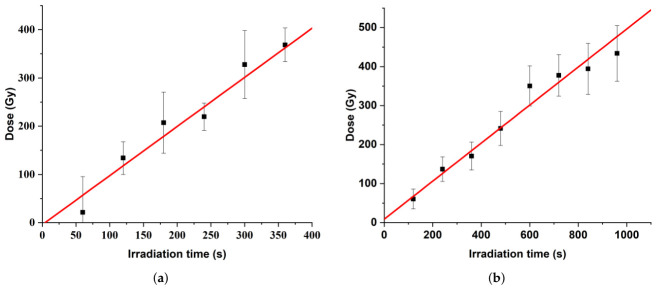
(**a**) The dependency of the dose absorbed by the Fricke solution upon electron beam irradiation on the irradiation time. (**b**) The dependency of the dose absorbed by the Fricke solution upon X-ray irradiation on the irradiation time.

Experiments showed that the doses absorbed by Fricke solution varied in the range 60–430 Gy for X-ray irradiation and in the range 21–368 Gy for electron beam irradiation. Figure 1 shows the dependency of the dose absorbed by the Fricke dosimeter on the exposure time of e-beam and X-ray irradiation. The dose rate was determined as the slope coefficient of the linear approximation of experimental data.

The dose rate absorbed by the Fricke solution during X-ray irradiation was $P_{X}=(0.48\pm 0.03)\;\mathrm{Gy}/s$ and $P_{e}=(1.01\pm 0.10)\;\mathrm{Gy}/s$ for electron beam irradiation.

To calculate the dose conversion coefficient, which is the ratio of the dose absorbed by water phantoms simulating wheat seeds to the dose absorbed by the Fricke dosimetry solution, we performed Geant4 computer simulation of Fricke solution and water phantom irradiation separately for electrons with the energy spectrum corresponding to the spectrum of UELR accelerator (Figure A1) and photons with the bremsstrahlung spectrum corresponding to the spectrum of X-ray tube set to perform at $U_{A}=80\;\mathrm{kV}$ (Figure A2).

**Figure A3:**
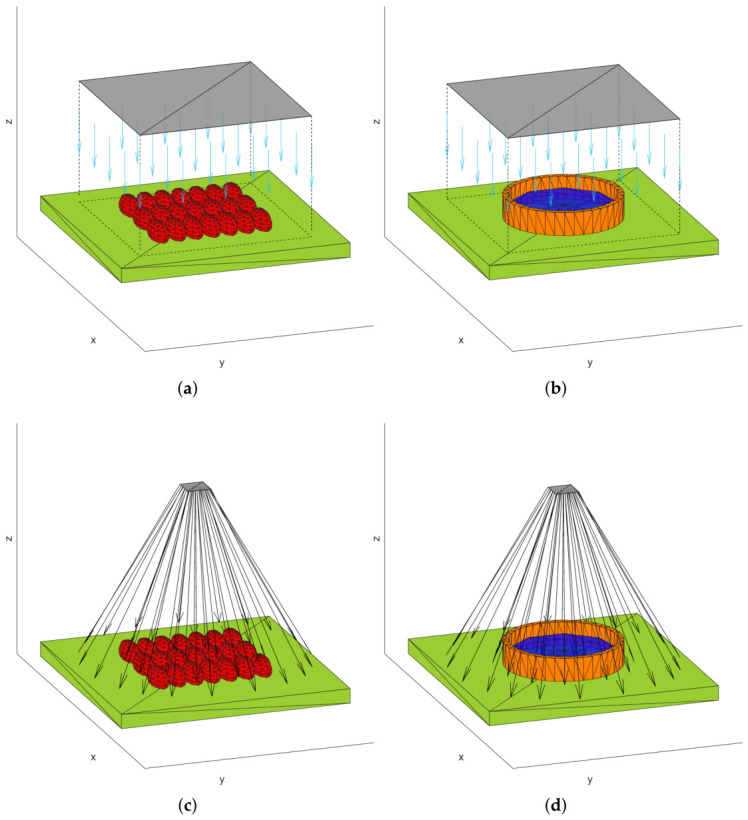
Geant4 simulation of the exposure of water phantoms representing: (**a**) e-beam irradiation of wheat seeds; (**b**) e-beam irradiation of Fricke solution; (**c**) X-ray irradiation of wheat seeds; (**d**) X-ray irradiation of Fricke solution. Aluminum plate is colored green, wheat seeds are red ellipsoids, X-ray and e-beam sources are gray squares, Petri dishes are colored orange and Fricke solution is colored blue.

The seeds (Figure A3) were simulated as water ellipsoids with semi-major axis a=1.5mm, intermediate axis b=3.5mm, and semi-minor axis c=2mm, having a density of $1.2\;g/cm^{3}$, which corresponds to the density of wheat seed. The ellipsoids were truncated along the intermediate axis in the third dimension to half the length of semi-minor half-axis in order to imitate the shape of the wheat seeds. The seeds were compacted tightly to form a 21 mm-long and 21 mm-wide rectangle, consisting of 3 × 7 ellipsoids. The Fricke solution in the Petri dish (Figure A3) was simulated as a shallow polypropylene cylinder with an outer radius of 17.5 mm, an inner radius of 16.5 mm, a base width of 1 mm, and a height of 10 mm. Fricke solution was simulated as water with the density of $1.024\;g/cm^{3}$, which corresponds to the density of Fricke solution. Fricke’s solution had the shape of a cylinder with a radius of 16.5 mm and a height of 2.2 mm.

During electron beam simulation, both the water phantoms and the Petri dish were placed on a 35 cm-long, 5.5 cm-wide, and 5 mm-thick aluminum parallelepiped simulating the base plate on which wheat seeds are placed for irradiation. The aluminum base plate with water phantoms located at 12 cm from the electron beam output was exposed to 10^7^ electrons. For X-ray simulation, a 20 cm-long, 15 cm-wide and 5 mm-thick aluminum base plate with water phantoms located at 12 cm from the beryllium window of X-ray tube was irradiated with 10^8^ photons.

Virtual detectors with the size and geometry corresponding to those of water phantoms simulating wheat seeds and Fricke solution recorded the absorbed energy in phantom. The dose absorbed by the water phantoms was calculated as the ratio of the total absorbed energy E to the mass of water phantoms. The total absorbed energy *E* is the sum of energies absorbed by the phantoms during interaction of each initial electron or photon. The dose absorbed by the simulated seeds and the dose absorbed by the simulated Fricke solution were determined using the following formula:

(2) $$D_{seed}=\frac{E_{seed}}{m_{seed}},$$

(3) $$D_{Fricke}=\frac{E_{Fricke}}{m_{Fricke}},$$

where $E_{seed}$ is the total energy absorbed by all water phantoms simulating wheat seeds, $m_{seed}$ is the total mass of the water phantoms simulating wheat seeds, $D_{seed}$ is the dose averaged across all phantoms simulating wheat seeds; $E_{Fricke}$ is the energy absorbed by the water phantom simulating Fricke solution, $m_{Fricke}$ is the mass of the water phantom simulating Fricke solution, and $D_{Fricke}$ is the dose absorbed by the water phantom simulating Fricke solution.

**Figure 2:**
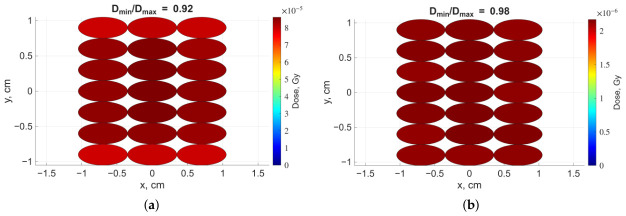
Average absorbed dose in water phantoms irradiated with: (**a**) e-beam; (**b**) X-ray.

Calculations have shown that during e-beam irradiation with 10^7^ electrons, the phantoms simulating seeds absorbed $8.32\times 10^{-5}\mathrm{Gy}$ and the Fricke solution absorbed $9.40\times 10^{-5}\mathrm{Gy}$. Thus, the dose conversion coefficient for electrons is $K_{e}=0.885$. Similarly, X-ray irradiation with $10^{8}$ photons resulted in $2.14\times 10^{-6}\mathrm{Gy}$ in the phantoms simulating the wheat seeds and $2.21\times 10^{-6}\mathrm{Gy}$ in Fricke solution. Thus, the dose conversion coefficient for photons is $K_{X}=0.968$. Computer simulation has shown that the seeds positioned centrally absorbed the maximum dose, with the exposure decreasing toward the periphery. The calculations have shown that the dose uniformity in the wheat seed layer irradiated with electrons is 0.92, while the seed layer exposed to X-rays is irradiated more uniformly with the dose uniformity of 0.98 (Figure 2).

Knowing the dose conversion coefficients and the dose rate absorbed by Fricke solution, the exposure time was adjusted to ensure that wheat seeds absorb the target doses $D_{seeds}$ for laboratory and field studies. Since the dose rate *P* absorbed by the object is proportional to electron beam current *I* both for electron beam irradiation and X-ray irradiation, the exposure time for e-beam or X-ray irradiation is calculated using the following formula:

(4) $$t=\frac{D_{seeds}}{KP}\frac{I_{Fricke}}{I},$$

where $D_{seeds}$ is the target dose, absorbed by wheat seeds during e-beam or X-ray irradiation, *I* is the beam current for wheat seed irradiation, $I_{Fricke}$ is the beam current for the Fricke solution, *K* is the dose conversion coefficient for e-beam or X-ray irradiation, *P* is the dose rate for the irradiation of the Fricke solution. Irradiation parameters for one-side X-ray irradiation for laboratory and field studies are presented in Table A1 and Table A2, respectively, and for electron beam irradiation in Table A3 and Table A4, respectively.

### 2.5. Computer Simulation of Absorbed Dose Distribution, Linear Energy Transfer Distribution and Radiation-Chemical Yield of ROS

The dose distribution and LET distribution throughout the irradiated wheat seeds were determined using the Geant4 toolkit [45]. The wheat seed was simulated as a water ellipsoid with semi-major axis a=1.5 mm, intermediate axis b=3.5 mm and semi-minor axis c=2 mm. The ellipsoids were truncated along the intermediate axis in the third dimension to half the length of semi-minor half-axis in order to imitate the shape of the wheat seeds. For electron beam irradiation, water ellipsoid was placed on a duralumin plate located at 12 cm from the beam output. During computer simulation, the water ellipsoid was irradiated with 10^7^ electrons having the energy spectrum represented in Figure A1. For X-ray irradiation, water ellipsoid placed on a polypropylene tray located at 12 cm from the beryllium window of X-ray tube was irradiated with $10^{8}$ photons having energy spectrum represented in Figure A2.

To calculate the absorbed dose distribution and LET distribution throughout the water ellipsoid irradiated with electrons or photons, the ellipsoid was divided by a virtual grid into 105 × 105 × 20 individual cubic cells. To map the dose in the ellipsoid, the dose *D* absorbed by each cubic cell was calculated using the following formula:

(5) $$D=\frac{\Delta E}{\Delta m},$$

where ΔE is the total energy transferred to the cubic cell, and Δm is the mass of the cubic cell.

For the edge cells of the water ellipsoid, the calculations were performed taking into account the difference in their mass. The margin of error $S_{i}$ of the absorbed energy value for each cubic cell was calculated by the following formula:

(6) $$S_{i}=\sqrt{\frac{1}{N_{i}-1}\sum_{j=1}^{N_{i}}E_{ij}^{2}-{\left(\frac{1}{N_{i}-1}\sum_{j=1}^{N_{i}}E_{ij}\right)}^{2}},$$

where $N_{i}$ is the number of events in the i-cell, $\sum_{j=1}^{N_{i}}E_{ij}$ is the total energy absorbed in the *i*-cell, and $\sum_{j=1}^{N_{i}}E_{ij}^{2}$ is the sum of the squares of the energies absorbed during Ni events in the *i*-cell. The dose calculated in each cell was normalized to the maximum value of the dose in the whole volume of the ellipsoid. The dose uniformity in the water phantom was calculated as the ratio of the minimum relative irradiation dose to the maximum dose value. The average LET value in the cell was calculated as follows:

(7) $$\bar{L}=\sum_{i}\frac{\Delta E_{i}}{\Delta l_{i}}w_{i},$$

where $\Delta E_{i}$ is the energy released by the *i*-th electron in the cell volume, $\Delta l_{i}$ is the track length of the *i*-th electron in the cell volume, and $w_{i}$ is the weighting factor determined using the following formula:

(8) $$w_{i}=\frac{\Delta l_{ij}}{\Delta_{lj}}.$$

Thus, the average LET value was determined as follows:

(9) $$\bar{L}=\frac{\sum_{i}\Delta E_{i}}{\sum_{i}\Delta l_{i}}.$$

The margin of error in the total energy absorbed in each cell did not exceed 2%.

For the calculation of the radiation-chemical yield of ROS, we used Geant4-DNA [46,47,48,49,50]. Geant4-DNA expands the capabilities of the Geant4 Monte Carlo simulation toolkit to address applications in radiation biophysics and radiobiology. Simulations in Geant4-DNA were carried out in sequential phases: the physical-chemical stage, which covers electron thermalization and the processes affecting ionized and excited water molecules, and the chemical stage, in which chemical species diffuse through the medium and undergo mutual reactions. In this study, we employed the online implementation of Geant4-DNA (version 11.3.2) to determine the G-values of ROS, generated by electrons and photons using the “chem6” example as the foundation for our calculations. The physics configuration included the G4EmDNAPhysics_option2 physics constructor and the G4EmDNAChemistry_option3 chemistry constructor. The physical-chemical stage was simulated up to 1 ps, while the chemical stage proceeded from 1 ps to 1 ms.

At first, the radiation-chemical yield *G* in terms of (Number of species)/(100 eV of deposited energy) and $\bar{L}$ were calculated as a function of initial electron energy, which allowed us to calculate the 3D radiation-chemical yield G distribution in units of (Number of species)/(100 eV of deposited energy) in wheat seeds. After that we multiplied $G(\bar{L})$ by the dose distribution to account its variances in spatial distribution, finally obtaining the radiation-chemical yield $G_{D}$ distribution:

(10) $$G_{D}=G\left(\bar{L}\right)D.$$

### 2.6. Laboratory Experiments on Biometric Properties and Phytopathogenic Status of Wheat Seeds After Irradiation

At the first year of the experiment, irradiated wheat seeds were delivered to the Siberian Federal Scientific Centre of Agro-BioTechnologies of the Russian Academy of Sciences for laboratory and field studies. In the laboratory, the wheat seeds were placed in Petri dishes on a Czapek Dox agar and germinated at a constant temperature of 20 ^∘^C [51]. On day seven after sowing, the germination of seeds was measured as the ratio of the number of germinated seeds to total number of seeds, and the length of the roots and seedlings was registered to assess the impact of pre-sowing e-beam and X-ray irradiation at different doses on the growth rate and biometric properties of plants [52]. In vitro growth of fungi was estimated by the average number and the average diameter of fungi colonies grown on Czapek Dox agar. The type of fungi was identified based on their morphological features, such as mycelium, spores, conidia, and other structures, using an optical microscopy. The number of fungi colonies of each species was estimated by counting the grown colonies in each Petri dish. The average diameter was estimated for all the fungal species. The experiment was repeated three times. Statistical processing of the obtained data was carried out using the SNEDECOR application software package [53] which uses standard mathematical processing methods.

### 2.7. Field Studies of Wheat Yield and Disease Rate After Pre-Sowing Irradiation

#### 2.7.1. Soil Conditions

The field studies were conducted over two years from May to September 2023–2024 in the soil and climatic conditions typical for the forest-steppe zone of Western Siberia. The soil of the experimental site was leached chernozem of medium loamy granulometric composition, pH = 6.7–6.8. The thickness of the humus horizon was 39 cm. Fractions of coarse dust 41–48% and fine sand 19–26% prevailed in all soil horizons. The density of the soil composition varied from $1.02\;g/cm^{3}$ in the arable layer to $1.46\;g/cm^{3}$. The density of the solid phase ranged $2.4–2.5\;g/cm^{3}$. In the meter-deep layer, the wilting humidity was within 109 mm, the lowest moisture capacity was 195 mm, and the total moisture capacity was 370 mm. The arable soil horizon had the following properties: 4.2–4.8% of humus, 0.27–0.41% of total nitrogen; easily mobile phosphorus amounted to 0.34–0.59 mg/kg of soil according to Karpinsky and Zamyatina and 18.0–18.5 mg/100 g according to exchangeable potassium amounted according to Chirikov [54,55] 7.0–7.7 mg/100 g of soil. According to [56], the soil in the forest-steppe zone of Western Siberia was contaminated with fungi *Bipolaris Sorokiniana*, *Alternaria* spp., and *Fusarium* spp.

In autumn, the soil was tilled to the depth of 18 cm. In spring, the soil was harrowed using a large tooth harrow to prevent moisture evaporation and then tilled to the depth of 10–15 cm. The repeatability of the experiment was six-fold with a seeding area of $6\;m^{2}$ per each dose sample. Each experimental section measured 1.0 m × 6.0 m, the distance between the rows was 15 cm from each other, and 90 seeds were sown in a meter-long row. The experiments were carried out using a three-row manual precision seeding machine. Crop care included treatments against weeds and pests [57]. Insecticide treatment was performed on June 6, June 20, and 15 July 2023, and on 14 June, 26 June, and 6 July 2024, using a mixture of water-dispersible granules Aktara (Syngenta, Basel, Switzerland) (thiamethoxam 250 g/kg), adjuvant Silvet 408 (UPL, King of Prussia, PA, USA) (100% modified heptamethyltrisiloxane), and antifoaming agent Penorex (Aqualar, Moscow, Russia) (dimethylsilinene).

#### 2.7.2. Climate Conditions

**Figure 3:**
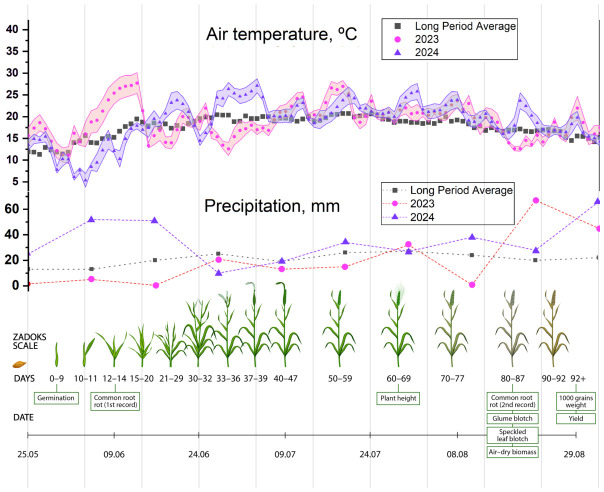
Weather conditions in 2023 and 2024 at the experimental site and phenological phases of wheat.

While in the year 2023, when the seeds were sown, the atmospheric temperature generally corresponded to the long period average, in 2024, the temperature was significantly higher than the long period average peaking in the second decade of May at 14 ^∘^C. From the third decade of May to the second decade of June, in 2023 the temperature significantly exceeded the long period average, reaching 27 ^∘^C at the peak in the first decade of June, which is 1.6 times higher than the long period average (Figure 3). To compare, the temperature at the same period in 2024 was lower than the long period average or generally corresponded to it. In 2024, the third decade of June was much warmer than the average with the temperature, reaching its maximum of almost 26 ^∘^C. While the temperature in July of 2023 and 2024 was on average 3–5 ^∘^C above the norm, from the second decade of August till the end of September 2023 and 2024, the temperature was on average 3–7 ^∘^C above the norm or corresponded to the long period average.

The precipitation over the two-year period of observation had a dramatically different pattern than the long period average, especially in spring and autumn. While in the year 2023, during the sowing and germination, the rainfall was completely absent, which required additional watering, in 2024 the precipitation was significantly higher than the long period average peaking in the first and second decade of June at 50 mm, which is 5 times higher than the norm. Both in 2023 and 2024, from the third decade of June to the third decade of July, when the formation of the main biomass takes place, the amount of the precipitation corresponded to the long period average. In 2023, following a decrease in precipitation in the first decade of August, there was a sharp increase in the amount of rainfall from 0 to 60 mm. Further from the second decade of August to the third decade of September, the precipitation exceeded the norm by 2–4 times. In 2024, from the third decade of August to the first decade of September, when grain filling occurs, the precipitation was 2–6 times higher than the long period average.

Since June 2023, when the seeds were planted and the plants started to develop and there was no precipitation, the wheat crop was irrigated on 2 and 4 June 2023 with 30 L of tap water per 6 m^2^ to ensure a more even germination of seedlings.

#### 2.7.3. Biometric Data, Quality Indicators, and Yield Parameters

The division of the wheat growth process into phenological stages was carried out according to the Zadox system [58]. Records and observations conducted from the beginning of June to the end of August 2023 and 2024 corresponded to the development stages of plants and included determining biometric data, productivity, and plant diseases. The germination rate calculated as the percentage of germinated seeds to those sown was measured on the seventh day after sowing.

Root rot caused by *Bipolaris sorokiniana* (Sorokin) Shoemaker (1959) and *Cochliobolus sativus* was evaluated on a four-point scale using the method of V.A. Chulkina [59], where the following hold:

- 0 points are the absence of signs of disease in all plant organs;
- 0.1 points are first signs of contamination when single light brown dots, small spots or stripes account for approximately 2.5% of the plant organ;
- 1 point is a low level of contamination when light or dark brown spots and stripes merge, making up about 25% of the plant organ;
- 2 points are a higher level of contamination when the affected dark brown or almost black tissue makes up approximately 50% of the plant organ;
- 3 points are a severe contamination when the affected dark brown or almost black tissue accounts for approximately 75% of the plant organ area;
- 4 points are the withering of the affected plant organ.

Root rot was measured twice on day 12–14 and day 80–87 after sowing. The wheat plants were shoveled to a depth of 20 cm from an area measuring 0.25 m × 0.25 m in the middle of each of the two rows, carefully shaken off from adhering soil particles and thoroughly rinsed. The degree of the root rot contamination was determined from freshly excavated samples. The air-dry biomass of 100 plants from the same crop were dried at room temperature and then weighted to assess the development of wheat on day 80–87 after sowing.

The height of the plants was registered on day 60–69 after sowing, with ten plants for the seeds irradiated with the same dose using the same irradiation source.

*Septoria* glume blotch and *Septoria* leaf blotch caused by *Septoria nodorum* was evaluated on a four-point contamination assessment scale on day 80–87 after sowing [60,61], where the following hold:

- 0 points are the absence of contamination of wheat leaves and ears;
- 0.1 points are the first signs of contamination of wheat leaves and ears;
- 1 point is a low level of contamination when one-fifth of the surface of leaves or/and single glumes are infected with *Septoria*;
- 2 points are a medium level of contamination when one-fifth to one-third of the surface of leaves or/and one-third of ear and single corns are infected with *Septoria*;
- 3 points are a high contamination when up to two=thirds of the surface of leaves or/and a half of ear and single corns are infected with *Septoria*;
- 4 points are a severe contamination when more than two-thirds of the surface of leaves or/and almost all glumes of the ear and all corns are infected with *Septoria*.

The rate of wheat contamination with *Septoria* was calculated for each plant organ and for each plant using the following formula [62]:

(11) $$R=\frac{\sum 100abn}{4N},$$

where *R* is the rate of wheat contamination measured in percentage terms, *a* is the number of plants with common attributes of the disease, *b* is the number of points corresponding to the four-point contamination assessment scale; *n* is the number of contaminated plants grown from the seeds irradiated with the same dose using the same irradiation source; and *N* is the total number of plants grown from the seeds irradiated with the same dose using the same irradiation source.

To assess the productivity of the wheat crop irradiated with the same dose using the same irradiation source, the plants were harvested from an area of 0.25 m × 0.25 m in the middle of each row on day 80–87 after sowing. The harvested biomass was air-dried at room temperature to a constant weight, and the resulting weight was expressed on a 100-plant mass basis. On day 92 after sowing, the grain yield in tons/ha was calculated on a 1000-grain mass basis, assuming the grains were harvested from an area of 0.25 m × 0.25 m in the middle of each row for the seeds irradiated with the same dose using the same irradiation source. Statistical processing of the obtained data was carried out using SNEDECOR V5 software and OriginPro 20018 9.5.1.195.

## 3. Results

### 3.1. Dose Distribution and Linear Energy Transfer (LET) in Wheat Seeds Irradiated with Accelerated Electrons and X-Rays

**Figure 4:**
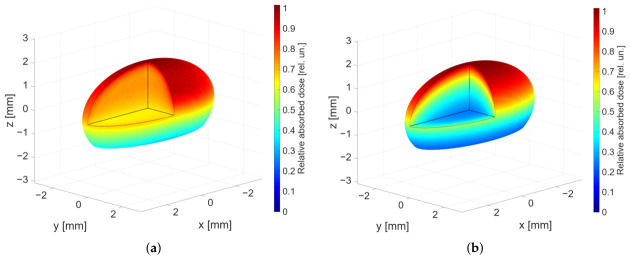
3D dose distribution in water phantoms irradiated with: (**a**) e-beam; (**b**) X-ray.

To get an insight into the dose distribution absorbed by the wheat seeds irradiated with e-beam and X-ray irradiation, we have simulated electron and X-ray irradiation of water phantoms representing the individual wheat seeds, factoring in the electron and X-ray one-side irradiation methods as well as the geometry and density of the seeds. As it can be seen from Figure 4, the maximum dose is absorbed by the surface layers of the water ellipsoids irradiated with electrons or X-rays, which is critical for the elimination of fungi located on the surface of the seeds. The dose uniformity, however, differs between the two irradiation methods when it comes to the irradiation of deeper layers of the seeds which are critical for germination; deeper layers of e-beam irradiated seeds are more uniformly exposed to irradiation than deeper layers of X-ray irradiated seeds. Figure 4 shows that 1 MeV electrons and 80 keV photons penetrate the whole volume of the seeds and the dose uniformity in the seeds irradiated with electrons is 0.4, which is higher compared to 0.2 for X-ray irradiation.

**Figure 5:**
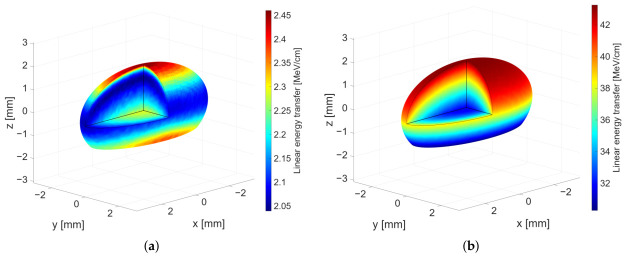
LET distribution in water phantoms irradiated with: (**a**) e-beam; (**b**) X-ray.

Recent studies [63,64,65,66] suggest that high LET irradiation is significantly more effective at inducing complex, clustered DNA damage, particularly double-strand breaks, inhibiting the DNA repair mechanism, as compared to low LET irradiation. Thus, the simulated LET distribution should be taken into account when evaluating the factors contributing to the difference in radiobiological effects of low-energy electrons and X-ray irradiation on the biometric parameters, phytopathogenic status of wheat seeds, plant diseases, and yield measured during the laboratory and field studies. As it can be seen from Figure 5, LET distribution in the phantoms differs significantly between e-beam and X-ray irradiation: while 1 MeV accelerated electrons account for relatively low LET values varying in the range 2.05–2.45 MeV/cm across the entire volume of the seed, X-ray irradiation causes LET values of secondary electrons occurring as a result of photoelectric effect and Compton effect to vary in range 30–45 MeV/cm. For X-ray irradiation, the LET distribution is similar to the dose distribution: the maximum LET value is reached on the surface of the seed, and it decreases towards the center of the seed. Since low-energy X-ray irradiation ensures relatively high LET values close to the surface of the seeds, it can be the preferred method for suppressing phytopathogens typically found on the surface of seed material, given the doses applied to seed irradiation are enough for suppressing fungi. Compared to X-ray irradiation, which ensures LET values at the level of 33–38 MeV cm in the deeper layers containing critical structures responsible for germination and growth of plant, LET values in the deeper layers of the seeds irradiated with accelerated electrons are more than 17 times lower at 2.1–2.2 MeV/cm. Such a dramatic difference between LET distribution depending on the type of irradiation can ensure different dynamics of biometrical parameters at different plant growth stages.

### 3.2. Impact of Irradiation on Germination, Root and Sprout Length of Wheat Revealed During Two-Year Laboratory Studies

#### 3.2.1. Biometrical Parameters

To assess the statistical significance of the impact of e-beam and X-ray irradiation on the biometrical parameters and phytopathogenic status of wheat seeds during the two-year laboratory studies, two-way ANOVA test was performed on non-irradiated and irradiated wheat seeds, taking into account the germination rate of seeds, length of roots and shoots, and the number and the diameter of fungi colonies found on the seeds. The doses and irradiation types applied to the wheat seeds were factored in the ANOVA test in order to estimate the impact of the doses and type of irradiation on the biometrical parameters and phytopathogenic status of the wheat seeds. To control the increase in the Type I error, the Bonferroni correction was applied, which sets the significance threshold α=0.05/10=0.005, where 10 is the total number of ANOVA tests performed. For any ANOVA test where the sample averages for different doses varied significantly with the *p*-value below the adjusted threshold of α=0.005, Tukey tests were performed within each type of irradiation with a significance threshold of α=0.05 to examine the differences between the parameters of irradiated and non-irradiated seeds. Further in the description, we are going to focus on the parameters of the irradiated seeds that differ significantly from the non-irradiated ones, marked with a “*” on the histograms. We also conducted sensitivity analysis with α=0.005 and power β=0.8 in G*Power 3 [67], resulting in minimal detectable effect size Cohen’s f=0.65 for the first year and f=0.83 for the second year. Our study is therefore capable of reliably detecting only substantial treatment effects. Consequently, statistically significant results observed in our ANOVAs likely represent biologically meaningful and robust treatment responses, as trivial effects would rarely be detected under these conditions. Conversely, non-significant findings should be interpreted as inconclusive rather than as evidence of no effect; true effects smaller than our detection threshold may exist but could not be resolved.

Overall, in the first and the second year of laboratory studies, the seed germination followed a similar pattern with a slight increase at the doses of 10–16 Gy by 7–13% from the values of the non-irradiated samples; the doses above 20 Gy either led to a decrease in the germination rate to a minimum level of 40% or its values practically remained constant. The germination rates of the non-irradiated seeds in the first and the second year were close with (88.4 ± 3.3)% and (90 ± 5.8)%, respectively.

**Figure 6:**
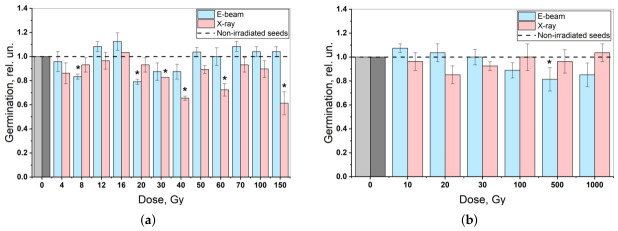
The dependency of the germination rate on the dose applied to the wheat seeds during electron beam irradiation (blue columns) and X-ray irradiation (pink columns) measured in: (**a**) 2023; (**b**) 2024. Samples that were significantly different from control are marked with “*”.

In the first year of laboratory studies, the dose dependency of the germination rate of the wheat seeds irradiated with accelerated electrons differed significantly from the germination rate of the wheat seeds irradiated with X-rays with $p=2.1\times 10^{-5}$ (Figure 6). The germination rate of the irradiated seeds differed significantly from the germination rate of the non-irradiated seeds with $p=1.7\times 10^{-13}$, and the interaction effect of the dose and type of irradiation on the germination rate was also significant with $p=2.2\times 10^{-10}$. E-beam irradiation with the doses of 8 Gy (p=0.011), and 20 Gy (p=0.0011) and X-ray irradiation with the doses of 30 Gy (p=0.010), 40 Gy ($p=2.8\times 10^{-7}$), 60 Gy ($p=1.8\times 10^{-5}$), and 150 Gy ($p=1.2\times 10^{-8}$) caused a significant decrease in the germination rate of seeds by 17–38% compared to the non-irradiated samples.

In the second year of laboratory studies, the effect of the type of irradiation was insignificant (p=0.67), the dose effect was also insignificant (p=0.17), but the interaction effect of the dose and type of irradiation was significant (p=0.0022). As it can be seen from Figure 6, only e-beam irradiation with the dose of 500 Gy led to a significant decrease in the germination rate by 18% (p=0.038).

Overall, in the first and the second year of laboratory studies, the wheat root length measured on day 7 after sowing decreased with the pre-planting irradiation dose exceeding 100 Gy irrespective of the irradiation type, which is evident in the second year of the study when a wider dose range up to 1000 Gy was studied. A two-year study revealed that a relatively low increase by 20% on average in the root length was registered after X-ray irradiation with the doses up to 100 Gy, which can be clearly seen in the first year of the research when the dose increment was relatively small. It should be noted that e-beam irradiation did not stimulate the growth of roots in either 2023 or 2024. The average root length of the plants grown from non-irradiated wheat seeds was (17.6±0.5) mm in the first year and (32.1±1.0) mm in the second year of the study.

**Figure 7:**
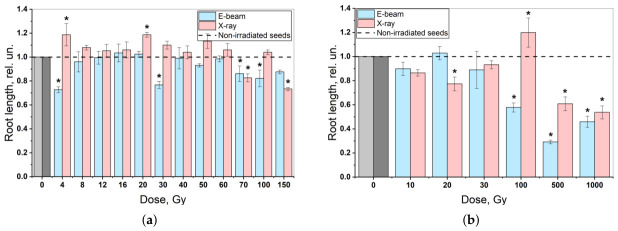
The dependency of root length on the dose applied to the wheat seeds during electron beam irradiation (blue columns) and X-ray irradiation (pink columns) measured in: (**a**) 2023; (**b**) 2024. Samples that were significantly different from control are marked with “*”.

While in the first year of laboratory studies the effect of the type of irradiation was noticeable ($p=6.2\times 10^{-20}$), the second year did not reveal a statistically significant difference (p=0.53) between the dose dependencies of the root length of plants grown from the seeds exposed to e-beam irradiation and X-ray irradiation (Figure 7). The root length of the plants grown from non-irradiated seeds differed significantly from the root length of the plants grown from irradiated seeds for the both years of the research, with $p=1.0\times 10^{-12}$ and $p=4.4\times 10^{-15}$ for the first and the second year, respectively. For the both years, the interaction effect of the dose and type of irradiation was also noticeable with $p=7.0\times 10^{-12}$ and $p=1.5\times 10^{-10}$ for the first and the second year, respectively. The comparison of the root length of the plants grown from the wheat seeds irradiated with e-beam irradiation over the two-year period has shown a steady decrease by up to 70% when the doses exceeded 70 Gy. The two-year study revealed that a steady decrease by up to 65% in the root length of the plants grown from the X-rayed seeds was registered when the doses exceeding 100 Gy were applied.

**Figure 8:**
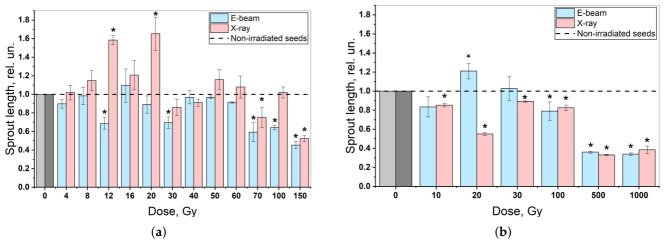
The dependency of sprout length on the dose applied to the wheat seeds during electron beam irradiation (blue columns) and X-ray irradiation (pink columns) measured in: (**a**) 2023; (**b**) 2024. Samples that were significantly different from control are marked with “*”.

The two-year study revealed that the doses of 10 Gy and below did not cause any significant change to the sprout length of the plants, irrespective of the irradiation type. The doses ranging 12–20 Gy caused the sprout length of plants to increase by 20–65% depending on the irradiation type and the year of research (Figure 8). The doses in the range of 40–60 Gy did not cause any significant change to the sprout length, and the further increase in the dose led to a dramatic decrease in the sprout length, irrespective of the irradiation type and the year of research. The average length of the sprouts grown from the non-irradiated seeds was (11.5±0.4) mm and (68.5±4.6) mm in the first and the second year, respectively.

In the first year of the study, X-ray irradiation with the doses ranging 12–20 Gy boosted the sprout length of the plants up to 65% (Figure 8). While the effect of the irradiation type on the sprout length of the plants was insignificant (p=0.40), the sprout length of the plants grown from irradiated seeds differed significant from the length of plants grown from non-irradiated seeds ($p=5.3\times 10^{-20}$), irrespective of the irradiation type, and the interaction effect of the dose and the irradiation type has significant impact on the sprout length of the plants ($p=5.7\times 10^{-12}$).

In the second year of the study, no doses which would considerably stimulate the growth of sprouts were registered. However, the e-beam irradiation dose of 20 Gy increased the sprout length up to 20% (Figure 8), which is close to the dose range which stimulated the growth of sprouts in the first year of the research. Similarly to the first year, while the effect of the irradiation type was not noticeable (p=0.0074), the dose effect was significant ($p=4.9\times 10^{-18}$), and the interaction effect of the irradiation type and the dose was also significant ($p=2.5\times 10^{-9}$).

#### 3.2.2. Phytopathogenic Status

Since the fungal contamination of wheat seeds has a negative impact on the quality and quantity of the yield, we studied to what extend the pre-planting irradiation with e-beam or X-rays with different doses inhibited the overall fungal contamination of the naturally infected seeds.

During the first year of the study, it was found that the non-irradiated seeds were primarily contaminated with fungi of the genus *Alternaria* that accounted for 84.4% of the total number of fungi. Apart from *Alternaria*, the fungi of the genus *Fusarium* and *Bipolaris* were detected on the surface of the seeds but their amount was relatively low, at 9.2% and 2.2%, respectively. Fungi causing seed mold belonged to *Aspergillus* and *Penicillium* and accounted for 2.2% and 2.0% of the total number of fungi of the genus, respectively. In the second year, the non-irradiated seeds were primarily contaminated with *Alternaria* (93.9%) and *Fusarium* (5.5%). The least represented group of fungi causing mold was fungi of the genus *Aspergillus* at 0.6%.

**Figure 9:**
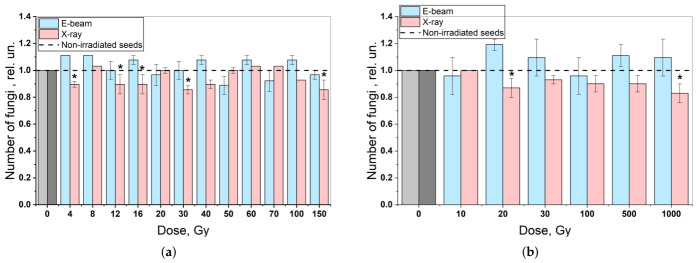
The dependency of the number of fungi on the dose applied to the wheat seeds during electron beam irradiation (blue columns) and X-ray irradiation (pink columns) measured in: (**a**) 2023; (**b**) 2024. Samples that were significantly different from control are marked with “*”.

As it can be seen from Figure 9, e-beam and X-ray irradiation with the doses up to 1000 Gy did not cause a statistically significant impact on the number of fungi, irrespective of the year of research. The number of fungi found on the irradiated seeds was close to the values of non-irradiated seeds and accounted for (9.5±0.3) and (8.5±0.2) in the first and the second year, respectively.

It should be noted that only X-ray irradiation with the selected doses of 4, 12, 16, 30, 40 and 150 Gy resulted in a significant decrease up to 14% in the number of fungi compared with the values of the non-irradiated seeds in the first year (Figure 9). In the second year, X-ray irradiation with the doses of 20 and 1000 Gy led to a significant decrease by 13–17% in the number of fungi compared with the control values (Figure 9). Such a decrease can be caused by a relatively high LET values of 35 MeV/cm of secondary electrons, occurring as a result of the photoelectric effect and Compton effect of interaction of X-rays with matter, on the surface of the seeds where fungi are typically found.

**Figure 10:**
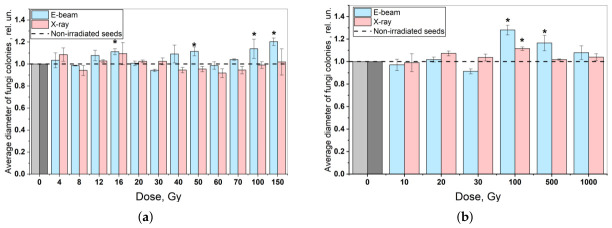
The dependency of the average diameter of fungi colonies on the dose applied to the wheat seeds during electron beam irradiation (blue columns) and X-ray irradiation (pink columns) measured in: (**a**) 2023; (**b**) 2024. Samples that were significantly different from control are marked with “*”.

The two-year study has revealed that neither low-energy electrons nor X-rays decreased the diameter of the colonies of fungi which infected the wheat seeds (Figure 10), due to the fact that such relatively low irradiation doses do not inhibit the activity of phytopathogens typically found on the surface of crops. It was found that the average diameter of fungal colonies found on the non-irradiated seeds was (31.2±0.7) mm in the first year and (30.3±0.9) mm in the second year, which can be explained by the similar levels of phytopathogen contamination of wheat seeds in 2023 and 2024. Elimination of phytopathogens on seed material would be achieved with the doses exceeding 1000 Gy [68]; however, such doses are not applicable to seeds as they would inhibit sprouting and crop yield. Interestingly, the selected doses of e-beam irradiation slightly increased the diameter of fungal colonies: the doses 100 Gy (p=0.0082) and 150 Gy ($p=8.0\times 10^{-5}$) increased the average diameter of fungi by 17–20% in the first year and even by 22% with the dose of 100 Gy ($p=2.3\times 10^{-5}$) in the second year. The relatively low stimulation on fungi growth can be caused by a combination of absorbed dose distribution and LET distribution in the surface layers of the seeds irradiated with accelerated electrons (Figure 4 and Figure 5). Compared to X-ray irradiation with the LET value of 42 MeV/cm, e-beam irradiation yields a relatively low LET value of 2.4 MeV/cm in the surface layers of wheat seeds, which can cause a stimulating effect on the growth of phytopathogens.

#### 3.2.3. Correlation Between Biometrical Parameters and Phytopathogenic Status

**Figure 11:**
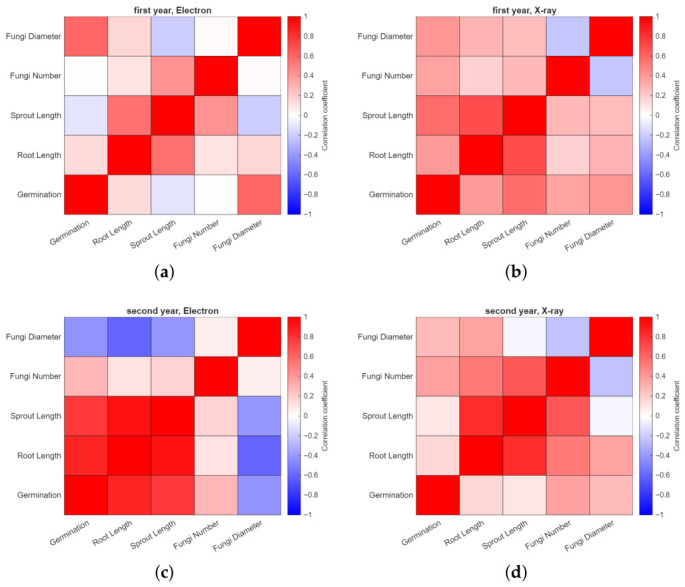
Correlation between the germination, root and sprout length, the number of fungi, and their average diameter in the non-irradiated wheat seeds and the wheat seeds irradiated with: (**a**) e-beam in 2023; (**b**) X-ray in 2023; (**c**) e-beam in 2024; (**d**) X-ray in 2024.

To gain a profound understanding of the impact of low-energy accelerated electrons and X-rays on biometrical parameters and phytopathogenic status of the wheat seeds, we have assessed the correlation between the germination, root and sprout length, the number of fungi and their average diameter in the non-irradiated wheat seeds and the wheat seeds irradiated with e-beam and X-ray irradiation studied in 2023 and 2024, which are shown on the heatmaps (Figure 11). A clear inverse relationship between the germination, root and sprout length of the seeds, and the number of fungi in the wheat seeds irradiated with electrons and their average diameter registered in the second year of the research (Figure 11) is a sign that even a slight increase in the fungal growth suppresses the plant growth. The two-year study has revealed that an increase in the seed germination has an overall beneficial effect on plant growth when the low-energy X-ray irradiation is applied (Figure 11).

### 3.3. Impact of Irradiation on Productivity and Plant Diseases of Wheat Revealed During a Two-Year Filed Study

To assess the impact of e-beam and X-ray pre-planting irradiation on the productivity and plant diseases of wheat grown from irradiated and non-irradiated seeds, which were sown in an open field in the climatic conditions typical for the forest-steppe zone of Western Siberia in 2023 and 2024, eight two-factor dispersion analyses (ANOVA) were performed on non-irradiated and irradiated wheat seeds taking into account germination, plant height, root rot and *Septoria* leaf and glume blotch, air-dry biomass and crop yield, as well as the weight of 1000 grains. The doses and irradiation types applied to the wheat seeds were factored in the ANOVA test in order to estimate the impact of the doses and type of irradiation on the productivity and wheat diseases. To control the increase in the Type I error, the Bonferroni correction was applied, which sets the significance threshold α=0.05/16=0.003125, where 16 is the total number of ANOVA tests performed. For any ANOVA test where the sample averages for different doses varied significantly with the *p*-value below the adjusted threshold of α=0.003125, Tukey tests were performed within each type of irradiation with a significance threshold of α=0.05 to examine the differences between the parameters of irradiated and non-irradiated seeds. Further in the description, we are going to focus on the parameters of the irradiated seeds that differ significantly from the non-irradiated ones, marked with a “*” on the histograms. Sensitivity analysis was also conducted with α=0.003125 and power β=0.8 in G*Power 3 [67], resulting in minimal detectable effect size Cohen’s f=0.86. Just like in laboratory studies, our study is therefore capable of reliably detecting only substantial treatment effects. Consequently, statistically significant results observed in our ANOVAs likely represent biologically meaningful and robust treatment responses, as trivial effects would rarely be detected under these conditions. Conversely, non-significant findings should be interpreted as inconclusive rather than as evidence of no effect; true effects smaller than our detection threshold may exist but could not be resolved.

**Figure 12:**
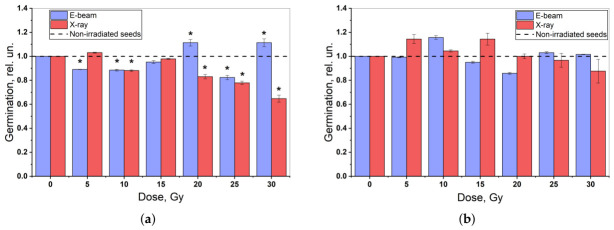
The dependency of germination rate on the dose applied to the wheat seeds during electron beam irradiation (violet columns) and X-ray irradiation (red columns) measured in: (**a**) 2023; (**b**) 2024. Samples that were significantly different from control are marked with “*”.

Figure 12 shows that while in 2023 pre-planting irradiation generally decreased the germination rate of wheat grown from irradiated seeds compared to the germination rate of wheat grown from non-irradiated seeds, irrespective of the radiation type, in 2024. no impact of pre-planting irradiation on the germination rate of wheat was observed. Considering that in 2024 the amount of precipitation at the germination stage was considerable, at around 30 mm, and while no rainfall was registered in the same period of 2023, it can be assumed that the absence of precipitation played a major role in suppression germination in 2023. The reference values for germination in the first year were (71.5 ± 1.05)%, and in the second—(25.2 ± 1.1)%.

In 2023, the impact of radiation type and the irradiation dose on the germination rate of wheat grown from irradiated seeds was noticeable with $p=2.78\times 10^{-16}$ and 1.21 × 10^−17^, respectively; the interaction effect of the radiation type and the dose was also significant ($p=2.84\times 10^{-22}$). It should be noted that e-beam irradiation increased the seed germination by 11% when they were irradiated with the doses of 20 Gy ($p=5.37\times 10^{-5}$) and 30 Gy ($p=5.37\times 10^{-5}$). X-ray irradiation negatively affected germination over the entire dose range, reducing it by 12–35%, when the seeds received the doses of 10 Gy ($p=3.25\times 10^{-6}$), 20 Gy ($p=4.94\times 10^{-8}$), 25 Gy ($p=1.42\times 10^{-7}$), and 30 Gy ($p=1.3\times 10^{-8}$). In 2024, the germination rate of the wheat grown from irradiated seeds did not differ significantly from the reference germination rate.

**Figure 13:**
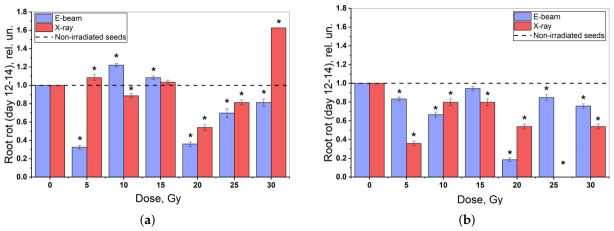
The dependency of the wheat root rot registered on day 12–14 after sowing the seeds on the dose applied to the wheat seeds during e-beam irradiation (violet columns) and X-ray irradiation (red columns) measured in: (**a**) 2023; (**b**) 2024. Samples that were significantly different from control are marked with “*”.

Overall, the two-year study suggests that pre-planting seed irradiation generally suppressed the proliferation of root rot, which was registered on day 12–14 after sowing the seeds, in wheat for both types of irradiation. While in 2023 the doses of 20 Gy and 25 Gy significantly decreased the proliferation of root rot in wheat irrespective of the radiation type (Figure 13), in 2024, all the doses in the range 5–30 Gy suppressed root rot in the wheat (Figure 13). The effect of radiation type and the dose was significant for both years of the experiment ($p=3.57\times 10^{-21}$ and 2.81 × 10^−28^ for the first year, and $p=1.49\times 10^{-21}$ and 2.76 × 10^−26^ for the second year, respectively) and the interaction effect of the dose and irradiation type was also noticeable with significance levels of $p=1.27\times 10^{-25}$ and 1.3 × 10^−24^ in 2023 and 2024, respectively.

**Figure 14:**
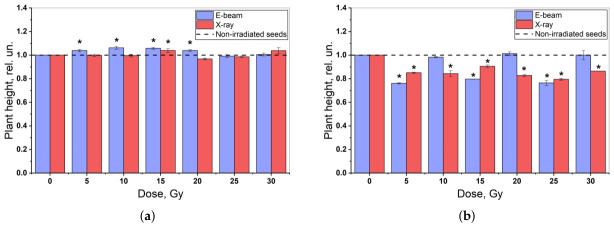
The dependency of the plant height registered on day 60–69 after sowing the seeds on the dose applied to the wheat seeds during e-beam irradiation (violet columns) and X-ray irradiation (red columns) measured in: (**a**) 2023; (**b**) 2024. Samples that were significantly different from control are marked with “*”.

In the first year of the experiment, the most effective e-beam irradiation dose inhibiting the root rot in wheat by 67% was 5 Gy with $p=3.68\times 10^{-10}$; in the case of X-ray irradiation, the dose of 20 Gy yielded the highest root rot inhibition rate of 46% ($p=1.67\times 10^{-9}$) (Figure 13). In the second year, the X-ray irradiation dose of 25 Gy was the most effective in terms of root rot inhibition with the root rot inhibition rate of 100% ($p=1.28\times 10^{-15}$) (Figure 13). E-beam irradiation with the dose of 20 Gy suppressed root rot in wheat by 80% ($p=4.78\times 10^{-12}$), which is the highest root rot inhibition rate among the whole e-beam irradiation doses applied to the seeds. However, the measurement of the root rot proliferation in the wheat performed 80–87 days after sowing showed that the spread of root rot in plants was 100% for all the plants studies including the plants grown from non-irradiated seeds. The two-year study showed that the effect of pre-planting irradiation on the root rot proliferation clearly manifests two weeks after sowing and is reduced to negligible values two month after pre-planting irradiation with low-energy e-beam or X-rays. It can be assumed that the rate of root rot in the wheat immediately before harvesting is determined by the level of soil contamination with the soil-borne fungus *Rhizoctonia*, *Fusarium* et al. While in the first year, the pre-planting irradiation with the doses in the range of 5–20 Gy slightly increased the height of the wheat plants measured on day 60–69 after sowing, in the second year, the wheat plants showed a considerable decrease in the height (Figure 14). However, the average height of the wheat grown from the non-irradiated seeds in the first year was (58.4±0.6) cm, which is considerably less than in the second year ((76.7±1.4) cm), which can be explained by a higher average atmospheric temperature in the range of 20–25 ^∘^C in 2024 compared to 11–17 ^∘^C in 2023.

In 2023, the maximum plant height was achieved with the e-beam irradiation doses 10 Gy ($p=8.4\times 10^{-5}$) and 15 Gy ($p=1.56\times 10^{-4}$) ensuring a 6% increase in the plant height (Figure 14). With the X–ray irradiation the maximum increase in the plant height was 4% when wheat seeds were irradiated with the dose of 15 Gy (p=0.0446).

In 2024, all the X-ray irradiation doses applied to the seeds decreased the wheat height, and the maximum decrease in the plant height was 15–20% at 5 Gy ($p=2.24\times 10^{-7}$), 10 Gy ($p=1.13\times 10^{-7}$), 15 Gy (p=0.01133), and 25 Gy ($p=3.45\times 10^{-7}$) (Figure 14). E-beam irradiation with the doses of 5 Gy ($p=1.27\times 10^{-7}$), 15 Gy ($p=5.9\times 10^{-8}$), and 25 Gy ($p=1.27\times 10^{-7}$) reduced the plant height by 20–24%.

During the two-year experiment, we studied the effect of pre-planting irradiation on the spread of *Septoria* leaf blotch and *Septoria* glume blotch detected on day 80–87 after sowing in order to determine the doses which enable plant growth and higher yield while suppressing fungal diseases in the plants. Overall, the wheat plants grown the non-irradiated seeds in 2023 were more contaminated with pathogen of leaf and stem diseases compared to 2024. While the rate of *Septoria* leaf blotch and *Septoria* glume blotch detected on the wheat grown from the non-irradiated seeds in 2023 was 100% and (91.7 ± 1.02)%, respectively, in the year 2024, the proliferation of these types of fungal diseases was considerably lower—(11.3±0.5)% and (10.6±1.1)%, respectively. Considering that the plants were grown in the same soil in both years of the research, the difference in the rate of soil-borne fungal diseases between these two years can be attributed to different weather patterns, especially the combination of rainfall and atmospheric temperature. In 2023, the level of fungal contamination of the soil was so high that neither e-beam nor X-ray irradiation had any effect on the suppression of the *Septoria* leaf blotch in the wheat grown from the irradiated seeds. In contrast, in 2024 the *Septoria* leaf blotch rate consistently decreased with an increase in the dose applied to the wheat seeds.

**Figure 15:**
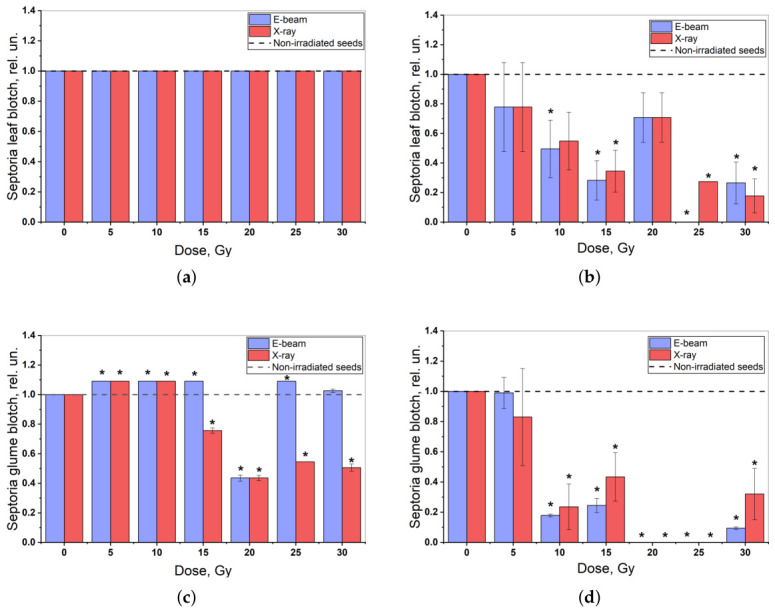
The dependency of the *Septoria* blotch rate registered on day 80–87 after sowing the seeds on the dose applied to the wheat seeds during e-beam irradiation (violet columns) and X-ray irradiation (red columns) of: (**a**) leaf blotch rate in 2023; (**b**) leaf blotch rate in 2024; (**c**) glume blotch rate in 2023; and (**d**) glume blotch rate in 2024. Samples that were significantly different from control are marked with “*”.

Compared to 2024, when pre-planting irradiation with the doses of 20 Gy and 25 Gy suppressed *Septoria* glume blotch almost completely, in 2023, the maximum reduction in the spread of *Septoria* glume blotch by 56% was achieved at the dose 20 Gy for both types of irradiation ($p=4.72\times 10^{-6}$ for e-beam and $p=3.57\times 10^{-12}$ and for X-ray irradiation) (Figure 15). In 2024, pre-planting irradiation with the doses exceeding 5 Gy significantly reduced the incidence of *Septoria diseases*. X-ray irradiation of 15 Gy (p=0.00778), 25 Gy (p=0.00327) and 30 Gy (p=0.00103) reduced the prevalence of *Septoria* leaf blotch by | 65–82%. Electron beam irradiation of 10 Gy (p=0.03154), 15 Gy (p=0.00193), 25 Gy (*p* = 6.52035 × 10^−5^), and 30 Gy (p=0.00154) suppressed *Septoria* leaf blotch by 45, 65, 73 and 82%. For both types of irradiation, the doses of 20 Gy ($p=5.3\times 10^{-5}$ for e-beam and $3.94\times 10^{-8}$ for X-rays) and 25 Gy ($p=5.3\times 10^{-5}$ for e-beam and $p=3.94\times 10^{-8}$ for X-rays) completely inhibited the development *Septoria* glume blotch.

**Figure 16:**
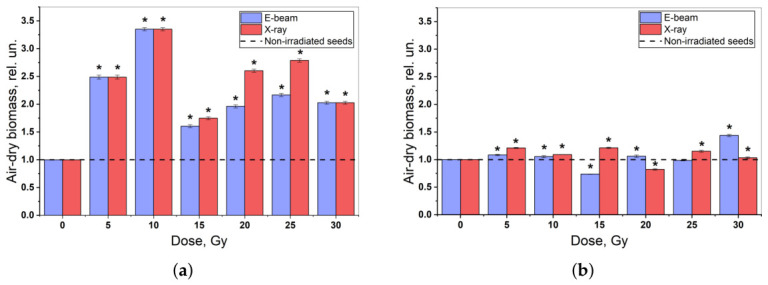
The dependency of the air-dry biomass registered on day 80–87 after sowing the seeds on the dose applied to the wheat seeds during e-beam irradiation (violet columns) and X-ray irradiation (red columns) measured in: (**a**) 2023; (**b**) 2024. Samples that were significantly different from control are marked with “*”.

The two-year field experiment has revealed that the response of the plants to the dose applied to the wheat seeds measured in air-dry biomass of the plants grown in 2023 was dramatically different from that in 2024. In 2023 the air-dry biomass of the plants grown from the seeds irradiated with electrons or X-rays was significantly heavier than the air-dry biomass of (202.5±5.3) g/100 plants plants from the plants grown from the non-irradiated seeds, especially at the dose of 10 Gy ensuring a 3.35-fold increase ($p=1.21\times 10^{-14}$ for accelerated electrons and $p=2.38\times 10^{-12}$ for X-ray irradiation) (Figure 16). On the contrary, in 2024 the amount of dry biomass increased only slightly with the maximum increase by 1.4 times at the dose of 30 Gy ($p=2.4\times 10^{-9}$) compared to the dry biomass of the plants grown from the non-irradiated seeds amounting to (485.5±3.4) g/100 plants (Figure 16).

**Figure 17:**
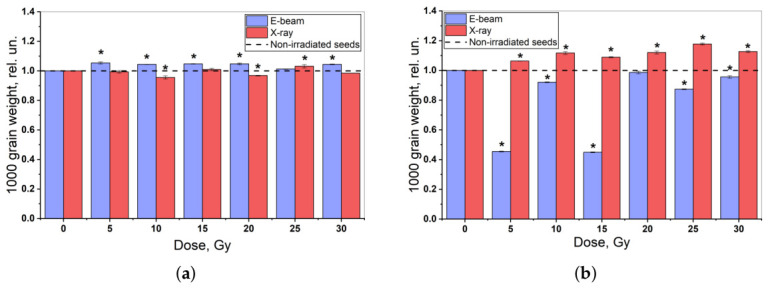
The dependency of the thousand grain weight air-dry biomass registered three months after sowing the seeds on the dose applied to the wheat seeds during e-beam irradiation (violet columns) and X-ray irradiation (red columns) measured in: (**a**) 2023; (**b**) 2024. Samples that were significantly different from control are marked with “*”.

According to the ISO 520-2014 standard [69] applicable to cereals and pulses, we used the weight of 1000 grains as the indicator of the crop yield quality. While in 2023 the difference between the 1000 grain weight in the yield grown from the irradiated seeds and the weight of 1000 grains in the reference yield was 3–5% (Figure 17), in 2024, e-beam irradiation decreased the 1000 grain weight in the yield by 5–55%, and depending on the irradiation dose, X-rays increased this indicator by 10% on average for all the doses applied (Figure 17). Thus, in the first year the reference weight of 1000 grains in the yield was (31.5±0.4) g and (36.50±0.12) g in the second year.

It should be noted that for both years of the study, the irradiation dose ($p=3.76\times 10^{-7}$ for 2023 and $1.09219\times 10^{-38}$ for 2024), the type of irradiation ($p=3.25\times 10^{-17}$ for 2023 and $2.13\times 10^{-35}$ for 2024), and the interaction between the dose and the irradiation type ($p=1.38\times 10^{-12}$ for 2023 and $5.42\times 10^{-38}$ for 2024) had a significant impact on the 1000 grain weight, which should be taken into account when pre-planting crop irradiation is implemented.

Having analyzed the crop yield throughout the two-year study, we have found that the doses in the range 5–15 Gy increased the yield in both years irrespective of the weather conditions. Therefore, it can be assumed that this dose range can increase the wheat yield without using agrochemicals.

**Figure 18:**
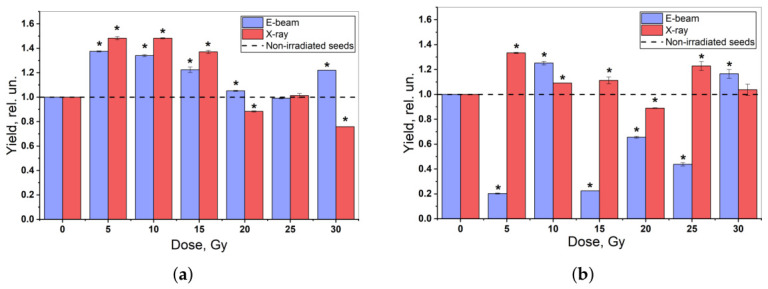
The dependency of the yield registered three months after sowing the seeds on the dose applied to the wheat seeds during e-beam irradiation (violet columns) and X-ray irradiation (red columns) measured in: (**a**) 2023; (**b**) 2024. Samples that were significantly different from control are marked with “*”.

In the first year of the experiment, an increase by 22–48% in the yield was observed when the seeds were irradiated with electrons and X-rays at the low doses of 5 Gy ($p=8.01\times 10^{-9}$ for electrons and $7.73\times 10^{-8}$ for X-rays), 10 Gy ($p=5.12\times 10^{-7}$ for electrons and 1.19 × 10^−9^ for X-rays) and 15 Gy ($p=2.22\times 10^{-7}$ for electrons and 9.01 × 10^−4^ for X-rays) (Figure 18).

In the second year, a 9–33% increase in the crop yield was also observed when the seeds were irradiated with X-ray irradiation at the doses of 5 Gy ($p=9.07\times 10^{-7}$), 10 Gy (p=0.00646) and 15 Gy ($p=9.72\times 10^{-4}$), as well as at the dose of 25 Gy ($p=2.69\times 10^{-7}$). On the contrary, accelerated electrons at the doses of 5 Gy ($p=5.04\times 10^{-14}$) and 15 Gy ($p=4.53\times 10^{-14}$) reduced the yield by 78–80% (Figure 18). Thus, X-ray pre-planting irradiation with the doses in the range of 5–15 Gy demonstrates a stable positive effect on the wheat yield. Electron beam irradiation with the dose of 10 Gy ($p=1.81\times 10^{-8}$) had a positive effect, as it increased the yield by 25%. Therefore, the dose of 10 Gy is seen as more predictable for increasing the wheat yield with the use of low-energy pre-planting irradiation.

The reference yield in the first year was higher compared to the second year amounting to 2.32 t/ha in the first year and (3.310±0.027) t/ha in the second year. The statistical data have shown that the dose ($p=5.58\times 10^{-38}$ and $1.02\times 10^{-24}$), the irradiation type ($p=5.08\times 10^{-11}$ and 2.91 × 10^−36^), and interaction between the dose and the type of irradiation ($p=5.89\times 10^{-30}$ and 9.9 × 10^−30^) had a significant impact on the yield. Therefore, the planning pre-planting irradiation planning stage should involve not only choosing the irradiation type and energy spectrum, but also determining the optimal dose range that increases the yield while reducing the plant diseases.

#### 3.3.1. Correlation Between Biometrical Parameters, Yield, and Plant Diseases

Since in 2023 and 2024 all the plants were infected with root rot measured on day 80–87 after sowing, this plant disease was not factored in the calculation of the correlation between the plant quality parameters. In 2023, all the plants were infected with *Septoria* leaf blotch, so this parameter was excluded from the calculation of the wheat quality correlation.

**Figure 19:**
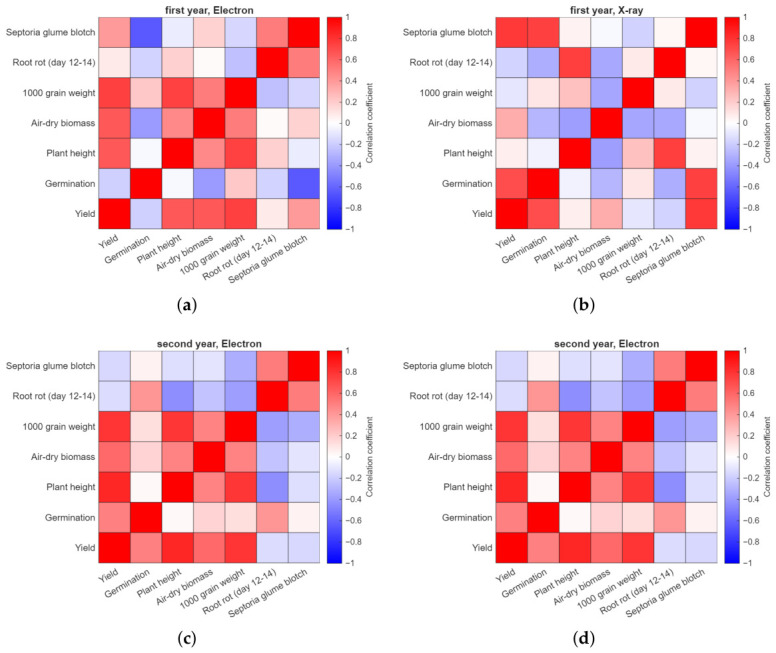
Correlation of wheat quality parameters for: (**a**) e-beam in 2023; (**b**) X-ray in 2023; (**c**) e-beam in 2024; (**d**) X-ray in 2024.

In both years of the study, the dose dependency of the air-dry biomass of plants had a relatively strong correlation with the dose dependency of amount of crop yield with the maximum the correlation coefficient of 0.87 for the plants grown from the seeds irradiated with the X-ray irradiation in 2024 (Figure 19).

Summarizing the results of the two-year study, it can be concluded that the dose dependency of the yield correlated well with the dose dependency of the germination rate with the maximum correlation coefficient of 0.7 for the plants grown from the seeds exposed to the X-ray irradiation in 2024; thus, the higher the germination rate, the higher the yield Figure 19). The electron irradiation of wheat seeds in 2023, on the other hand, led to a slightly negative correlation between the yield and the germination rate, since the electron irradiation doses which slightly suppressed the germination of the wheat had a positive impact on the yield.

The data show that in both years the height of the plants grown from the seeds exposed to electrons, which were measured during the flowering phase, correlated well with the 1000 grain weight (0.65 in 2023 and 0.85 in 2024) and the total yield (0.74 in 2023 and 0.79 in 2024), since a slight increase in the plant height in 2023 led to an increase in the wheat productivity, and conversely, a decrease in the plant height in 2024 caused the yield and the 1000 grain weight to decrease for most of the doses applied.

A traceable connection between plant diseases and the yield differed between the years depending on the type of irradiation. When the seeds were exposed to X-ray irradiation, the correlation between the root rot in plants and the yield was negative since the suppression of root rot was associated with an increase in the yield and the amount of air-dry biomass.

The extend of the damage of *Septoria* glume blotch has a clear correlation with the grain weight in the crop, since the suppression of *Septoria* glume blotch was associated with an increase in 1000 grain weight in 2023 and 2024 for both types of irradiation. When the seeds were irradiated with accelerated electrons in the first year of the study, the presence of root rot and *Septoria* glume blotch negatively affected the seed germination and grain weight.

#### 3.3.2. Optimal Dose Ranges for Wheat Irradiation with Low-Energy Accelerated Electrons and X-Rays

**Figure 20:**
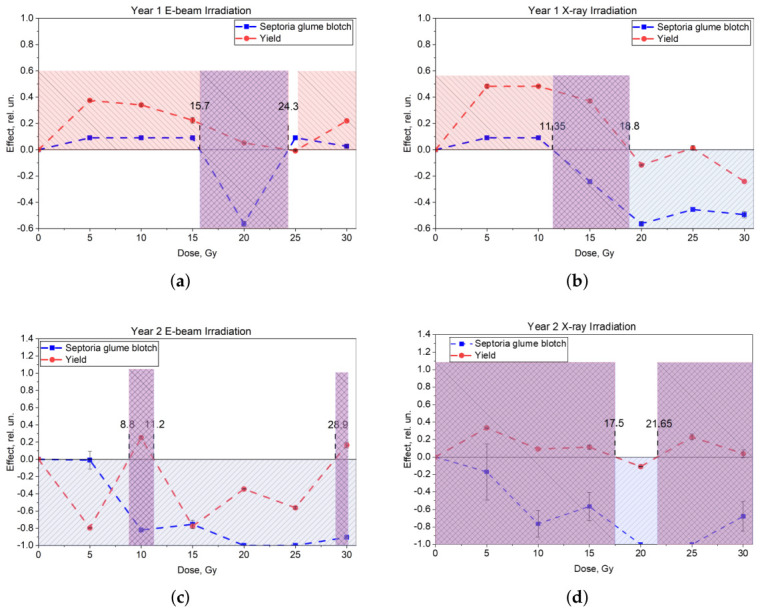
Dose dependencies of the yield (red curve) and *Septoria* glume blotch rate (blue curve) for the plants grown from the seeds irradiated with: (**a**) e-beam in 2023; (**b**) X-ray in 2023; (**c**) e-beam in 2024; and (**d**) X-ray in 2024. Dose intervals that increase the yield are colored in red, dose intervals that suppress glume botch rate are colored in blue.

Since the goal of pre-planting irradiation is to increase the yield and its quality, the established optimal dose of low-energy e-beam irradiation and X-ray irradiation of wheat seeds should be able to increase the yield and suppress plant diseases. To determine the optimal doses of pre-planting wheat irradiation we plotted the dose curves of wheat yield and the *Septoria* glume blotch rate against the reference parameters of the plants grown from the non-irradiated seeds (Figure 20).

In 2023 while most doses of e-beam pre-planting irradiation caused the yield to increase by 10–40%, the only dose that suppressed *Septoria* glume blotch by almost 40% was 25 Gy; therefore, it can be assumed that 25 Gy is the optimal for e-beam irradiation (Figure 20) if the goal is to enhance the wheat quality and the dose range 5–15 Gy is the optimal for increasing the yield. X-ray pre-planting irradiation with the dose of 15 Gy increased the yield by 40% (Figure 20) while suppressing *Septoria* glume blotch by almost 40%. Similarly, when the seeds were exposed to X-ray irradiation a significant increase in the yield was registered when the doses in the range of 5–15 Gy were applied.

In 2024, when the climate conditions, in particular, the amount of rainfall and temperature were significantly different, the optimal irradiation doses were slightly different from the doses determined as optimal in 2023. While the e-beam irradiation dose of 10 Gy was the most effective for increasing the yield and the suppression *Septoria* glume blotch (Figure 20), all the X-ray irradiation doses except 20 Gy increased the yield by 10–25% and at the same time reduced the rate of plant diseases by 10–100% (Figure 20).

Since the optimal doses for pre-planting wheat irradiation appear to be different in 2023 and 2024, it can be concluded that the efficiency of pre-planting irradiation is tightly linked with the climate conditions. The two-year comparison of the two irradiation types has revealed that low-energy X-ray pre-planting irradiation is more consistently effective in terms of increasing the wheat yield and suppressing fungal diseases in the wheat plants. Having analyzed the two irradiation types during the two-year study, it can be concluded that the dose range 5–15 Gy is more preferable for pre-planting wheat irradiation.

## 4. Discussion

To compare the results of our research with the precedents, we have reviewed the literature available to date. The previous studies on pre-planting wheat seed irradiation conducted by the global scientific community used gamma irradiation generated by ^137^Cs or ^60^Co radioisotopes to boost germination, increase biomass, and enhance the crop yield [70]. Overall, the dose range 5–15 Gy, considered to be optimal in our study as it increases the yield making plants more resistant to fungal diseases, aligns well with the doses obtained by others. For example, the gamma irradiation dose of 20 Gy is recommended in the study [71] as it increased the number and length of roots both in laboratory and greenhouse conditions. Similarly, in our laboratory study, the low-energy e-beam irradiation doses of 10–16 Gy slightly increased the germination rate by 7–13%. The doses ranging from 12–20 Gy caused the sprout length of plants to increase by 20–65% depending on the irradiation type and the year of research.

A review of the literature indicates that a further increase in the irradiation dose led to a reduction of biometrical parameters, such as length of roots, sprouts, and biomass. In [72], for instance, an increase in the irradiation dose up to 400 Gy did not cause a significant change in the wheat seed germination rate under laboratory conditions, which is consistent with our laboratory results of the impact of accelerated electrons and X-ray irradiation in the dose range of 4–150 Gy on the germination rate of wheat seeds. In [73], gamma irradiation with the doses of 200–300 Gy reduced the length and biomass of plants. In our two-year laboratory study, the dose exceeding 100 Gy decreased the wheat and sprout root length irrespective of the irradiation type.

On the whole, the field studies conducted by our team have yielded less predictable results in view of the fact that outdoor cultivation of wheat is affected by a wide variety of different factors, such as soil conditions, weather patterns, fungal contamination of soil. Thus, while in 2023 X-ray irradiation with the doses 5–15 Gy increased the wheat yield up to 50%, in 2024 only the dose of 5 Gy had a noticeable effect on the yield, causing it to increase by 30% in the same soil. In 2023 the air-dry biomass of the plants grown from the irradiated seeds was significantly heavier than the air-dry biomass of reference plants, especially at the dose of 10 Gy ensuring a 3.35-fold increase. On the contrary, in 2024 the amount of air-dry biomass increased only slightly with the maximum increase by 1.4 times at the dose of 30 Gy. To compare [74], gamma-irradiated wheat seed in with the dose of 10–100 Gy in a field experiment increased the biomass of plants by 3–4 times and the number of shoots by 2.4–3.4 times. The maximum crop yield 286% of the control value was achieved when the crop was treated with the dose of 70 Gy.

To gain a deeper understanding of the stimulation effect of low-dose pre-planting seed irradiation and the difference of the impact of e-beam and X-rays on biometric properties, crop yield, and plant diseases, it is essential to consider the mechanisms that trigger hormesis in plants as it causes an adaptive stress response in plants after irradiation.

The impact of irradiation on biological tissues as a result of both direct ionization of atoms and molecules and indirect action through the radiolytic species lead to biological dose-effects in biomacromolecules. A complex set of events following irradiation is typically divided into three stages. Stage one involves initial deposition of energy and formation of spurs within $10^{-16}$ s. Stage two is thermal equilibrium achieved within $10^{-12}$ s which gives rise to stable molecules and radicals, such as aqueous electrons and $H_{2}O^{+}$ radicals. During stage three, radicals $H_{3}O^{+}$, $H_{2}$, $H_{2}O_{2}$, $e_{\mathrm{aq}}^{-}$, $H^{•}$, and ^•^OH diffuse away from their sites of formation, and they react until all spur processes are completed within $10^{-6}$ s [75]. These radicals cause lipid peroxidation and oxidative modification of proteins and nucleic acids as a result of oxidative stress in the cells [76]. While high irradiation doses lead to the formation of large amounts of reactive oxygen species involved in oxygen-dependent reactions which lead to radiation-induced degradation of nucleic acid bases and lipids, low irradiation doses lead to the activation of mechanisms that ensure the stability and adaptability of cells caused by non-specific biochemical reactions of cells to mild physical exposure [77].

Recent studies suggest that a slight increase in ROS levels enhances the regulation of genes responsible for regulating the antioxidant defense system, including enzymatic antioxidants such as superoxide dismutase, peroxidase, and catalase, as well as non-enzymatic antioxidants such as glutathione and ascorbate [78]. It has been established that an increase in enzyme activity leads to a higher rate of seed germination, which makes seeds more resistant to the change in environmental conditions [79]. There are studies indicating a change in the transcriptional activity of genes encoding phytohormone metabolism [80,81], which leads to an increase in the concentration of hormones that accelerate crop growth and to a decrease in hormones that inhibit plant growth.

It should be noted that irradiation with low doses increase the permeability of the seed shell to water and other nutrients [82], thereby accelerating the process of hydration between the cell wall and protoplasm, initiating metabolic processes for the accumulation of proteins, carbohydrates, phenols, flavonoids, phytohormones, and other substances, as well as increasing photosynthesis and stomatal conductance, which leads to an improvement in seed germination and an increase in crop yields. Recent studies suggest that low doses of radiation can accelerate cell mitosis, resulting in an increase in plant biomass [83].

Since electrons and photons interact differently with biological tissues, which is manifested in the different dose distributions in the wheat seeds irradiated with electrons and photons, different radiobiological effects of pre-planting irradiation with accelerated electrons and X-rays can be explained by the difference in the distribution of ionization events, LET values, and radiation-chemical yield of ROS throughout the entire volume of irradiated seeds.

**Figure 21:**
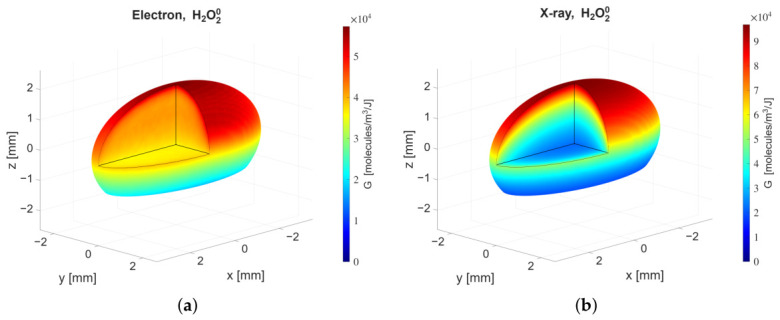
The 3D distribution of the radiation-chemical yield of $H_{2}O_{2}$ in water phantom irradiated with: (**a**) 1 MeV electrons; (**b**) 80 keV photons.

It is known from the literature that hydrogen peroxide $H_{2}O_{2}$, which occurs during seed irradiation through water radiolysis and subsequent ROS reactions, is a key signaling mediator that links primary exposure to radiation with the long—term adaptive response of the plant. It is through the action of hydrogen peroxide $H_{2}O_{2}$ that the effect of hormesis is largely realized [84]. Figure 21 shows the distribution of the radioionic chemical yield of $H_{2}O_{2}$ radical throughout the volume of the water phantom emitting a seed, calculated using Geant4-DNA to detect the difference between the action of ROS initiated by low-energy electrons and photons both in the surface and interior layers of water phantom. It should be noted that radiation-chemical yields for $H_{2}O_{2}$ during e-beam and X-ray irradiation are close in values, and the distributions of the radiation-chemical yield correlate well with the absorbed dose distributions. For X-ray irradiation, while the maximum of the G-value for $H_{2}O_{2}$ amounts to $9.8\times 10^{4}$ molecules/$m^{3}$/Joule in the surface layers of the water phantom where seed coat can be found, internal structures responsible for germination are attacked by $H_{2}O_{2}$ radicals with a yield five times lower (Figure 21). For 1 MeV e-beam irradiation, the distribution of $H_{2}O_{2}$ G-value is more uniform compared to X-ray irradiation, and the internal structures are attacked by $H_{2}O_{2}$ radicals with the yield of $3.7\times 10^{4}$ molecules/$m^{3}$/Joule, which is two times higher compared to electrons (Figure 21).

The analysis of an increase in biometric parameters measured during laboratory studies suggests that a higher efficiency of pre-planting X-ray irradiation compared to e-beam irradiation is achieved due to a relatively low radiation-chemical yield of $H_{2}O_{2}$ in the area of internal structures responsible for crop germination, which is confirmed by a more stable effect in increasing the yield and reducing the rate of *Septoria* diseases.

## 5. Conclusions

The two-year laboratory and field study involving the comparison of the results of the pre-planting irradiation of wheat seeds with 1 MeV accelerated electrons and 80 keV X-rays has shown that the dose range 5–15 Gy is more preferable for pre-planting wheat irradiation, irrespective of the type of irradiation, and low-energy X-rays ensure a higher yield and make wheat plants more resistant to Septoria diseases compared to low-energy electrons.

The two-year field study has established that the result of pre-planting irradiation depends not only on the irradiation parameters but also on the climate conditions, including atmospheric temperature and precipitation level. It has been found that the effect of pre-planting irradiation was more pronounced in the year 2023, which was marked by a combination of elevated temperatures and a lack of precipitation compared to the average during the first month after sowing: while in 2024, when the precipitation rate was higher than the average and the temperature fluctuated around the average values, the maximum increase in the crop yield was up to 25%, in the year 2023, the crop yield increased up to 50%.

The study suggests a comprehensive approach to pre-planting seed irradiation which would enhance the accuracy of the irradiation parameters for maximizing the yield and crop quality considering the selected irradiation method and individual properties of seeds. The novelty of the approach consists of the use of Geant4 and Geant-DNA computer simulation to estimate the irradiation dose and LET distribution, as well as the distribution of the radiation-chemical yield of radicals in seeds in order to make the results of pre-planting irradiation more predictable by factoring in the relationship between the effect of pre-planting irradiation and the distribution of irradiation parameters throughout the entire volume of irradiated seeds.

Since recent studies indicate reactive oxygen species play an important role in triggering biochemical processes responsible for increasing the germination rate, biomass of plants, and crop yield, our further research will investigate the impact of low doses of different types of low-energy irradiation on a complex molecular network that activates genes responsible for the enzymatic and non-enzymatic antioxidant defense systems and the genes encoding phytohormone metabolism.

## Figures and Tables

**Table 1:** Summary of seed allocation.

Type of Study	Laboratory		Field	
Year	2023	2024	2023	2024
Radiation type	X-ray/e-beam			
Dose, Gy	0, 4, 8, 12, 16, 20, 30, 40, 50, 60, 70, 100, 150	0, 10, 20, 30, 100, 500, 1000	0, 5, 10, 15, 20, 25, 30	
Seeds per dose	30		95	
Seeds per year	780	420	1330	1330
Total number of seeds	1200		2660	

## Data Availability

Data are available within the article, Appendix A, and upon request from the corresponding author.

## Appendix A

**Table A1:** X-ray irradiation parameters for laboratory studies.

$D_{\mathrm{seeds}}$, Gy	Margin of Error, Gy	Beam Current, mA	Exposure Time, s
4.00	0.25	1.00	42.39
8.00	0.49	1.00	84.77
12.00	0.74	1.00	127.16
16.00	0.98	1.00	169.54
20.00	1.23	1.00	211.93
30.00	1.85	1.00	317.89
40.00	2.46	1.00	423.85
50.00	3.08	1.00	529.82
60.00	3.69	1.00	635.78
70.00	4.31	1.00	741.74
100.00	6.15	1.00	1059.6
150.00	9.23	4.00	397.36
500.00	30.77	4.00	1324.54
1000.00	61.54	4.00	2649.08

**Table A2:** X-ray irradiation parameters for field studies.

$D_{\mathrm{seeds}}$, Gy	Margin of Error, Gy	Beam Current, mA	Exposure Time, s
5.00	0.31	5.00	10.60
10.00	0.62	5.00	21.19
15.00	0.92	5.00	31.79
20.00	1.23	5.00	42.39
25.00	1.54	5.00	52.98
30.00	1.85	5.00	63.58

**Table A3:** Electron beam irradiation parameters for laboratory studies.

$D_{\mathrm{seeds}}$, Gy	Margin of Error, Gy	Beam Current, mA	Exposure Time, s
4.00	0.39	0.03	29.59
8.00	0.79	0.03	59.19
12.00	1.18	0.03	88.78
16.00	1.57	0.03	118.38
20.00	1.96	0.03	147.97
30.00	2.95	0.03	221.95
40.00	3.93	0.03	295.94
50.00	4.91	0.03	369.92
60.00	5.89	0.03	443.91
70.00	6.88	0.03	517.89
100.00	9.82	0.20	110.98
150.00	14.73	0.20	166.47
500.00	49.11	0.20	554.88
1000.00	98.21	0.20	1109.77

**Table A4:** Electron beam irradiation parameters for field studies.

$D_{\mathrm{seeds}}$, Gy	Margin of Error, Gy	Beam CURRENT, mA	Exposure Time, s
5.00	0.49	0.02	55.49
10.00	0.98	0.02	110.98
15.00	1.47	0.03	110.98
20.00	1.96	0.03	147.97
25.00	2.46	0.03	184.96
30.00	2.95	0.03	221.95
